# Phosphorylation of the aggregate-forming protein alpha-synuclein on serine-129 inhibits its DNA-bending properties

**DOI:** 10.1016/j.jbc.2021.101552

**Published:** 2021-12-30

**Authors:** Sydney E. Dent, Dennisha P. King, Valerie R. Osterberg, Eleanor K. Adams, Marilyn R. Mackiewicz, Tamily A. Weissman, Vivek K. Unni

**Affiliations:** 1 Department of Neurology, Jungers Center for Neurosciences Research, Oregon Health & Science University, Portland, Oregon, USA; 2 Department of Chemistry, Portland State University, Portland, Oregon, USA; 3 Department of Biology, Lewis & Clark College, Portland, Oregon, USA; 4 OHSU Parkinson Center, Oregon Health & Science University, Portland, Oregon, USA

**Keywords:** alpha-synuclein, Parkinson disease, DNA-binding protein, phosphorylation, aSyn, alpha-synuclein, DSb, DNA double-strand break, EMSA, electrophoretic mobility shift assay, NAC, nonamyloid beta-component, WT, wild-type

## Abstract

Alpha-synuclein (aSyn) is a vertebrate protein, normally found within the presynaptic nerve terminal and nucleus, which is known to form somatic and neuritic aggregates in certain neurodegenerative diseases. Disease-associated aggregates of aSyn are heavily phosphorylated at serine-129 (pSyn), while normal aSyn protein is not. Within the nucleus, aSyn can directly bind DNA, but the mechanism of binding and the potential modulatory roles of phosphorylation are poorly understood. Here we demonstrate using a combination of electrophoretic mobility shift assay and atomic force microscopy approaches that both aSyn and pSyn can bind DNA within the major groove, in a DNA length-dependent manner and with little specificity for DNA sequence. Our data are consistent with a model in which multiple aSyn molecules bind a single 300 base pair (bp) DNA molecule in such a way that stabilizes the DNA in a bent conformation. We propose that serine-129 phosphorylation decreases the ability of aSyn to both bind and bend DNA, as aSyn binds 304 bp circular DNA forced into a bent shape, but pSyn does not. Two aSyn paralogs, beta- and gamma-synuclein, also interact with DNA differently than aSyn, and do not stabilize similar DNA conformations. Our work suggests that reductions in aSyn’s ability to bind and bend DNA induced by serine-129 phosphorylation may be important for modulating aSyn’s known roles in DNA metabolism, including the regulation of transcription and DNA repair.

Alpha-synuclein (aSyn) is a 140 amino acid–long protein found in vertebrates, where it is known to localize to the presynaptic nerve terminal (1, 2, 3) and nucleus (1, 4, 5, 6, 7, 8, 9). aSyn is linked to genetic forms of parkinsonism in families with an autosomal dominant inheritance pattern. The first such point mutation found (A53T) was in the N-terminal phospholipid-binding domain (10). Since then, five additional point mutations (A30P, E46K, H50Q, G51D, A53E) have been suggested to cause parkinsonism (11, 12, 13, 14, 15, 16, 17), and all occur within a 24 amino acid stretch of the N-terminal phospholipid-binding domain, indicating the potential importance of this region in triggering disease. This initial genetic linkage between aSyn and parkinsonism led to the discovery that the pathological hallmark of sporadic (nongenetic) Parkinson’s Disease, the Lewy body, is composed primarily of misfolded aSyn (18), in a combination of fibrillar and granular aggregation states (19, 20). A subset of prominent neurodegenerative conditions in addition to Parkinson’s Disease, including Dementia with Lewy bodies, Multiple System Atrophy, and Pure Autonomic Failure, are typified by the formation of aSyn aggregates. This has led to the adoption of the term “synucleinopathy” to describe the group of diseases characterized by pathological aggregation of aSyn. Interestingly, increased expression of aSyn has been shown to occur in certain cancers, most prominently in the skin cancer melanoma (21, 22, 23), a cancer with increased incidence in Parkinson’s Disease patients (24, 25, 26, 27, 28, 29).

aSyn’s roles within the presynaptic terminal have been extensively explored and multiple specific functions in regulating chemical neurotransmission within this subcellular compartment have been described, including modulating exocytosis (30, 31), endocytosis (32, 33), SNARE complex assembly (34), and vesicle clustering (35, 36, 37). Several studies have shown that aSyn prefers binding highly curved, negatively charged phospholipid membranes through its N-terminal domain that associates with the outer leaflet of the convex-curved phospholipid bilayer of synaptic vesicles by adopting a complementary, concave-curved alpha-helical structure composed of two antiparallel helices (38, 39, 40). It is through these interactions with neurotransmitter vesicles that aSyn is thought to mediate its presynaptic functions.

In addition to its functions in the presynaptic terminal, aSyn can also localize to the nucleus where it is less well studied but has been suggested to modulate several DNA- and RNA-dependent processes. These include regulation of histone modification (4, 41), transcription (42, 43), rRNA (44) and mRNA (45, 46) metabolism. Our recent work also suggests a previously unrecognized role for aSyn in modulating DNA double-strand break (DSB) repair (9). Many of these potential functions are dependent on aSyn’s ability to bind double-stranded nucleic acids, including DNA. Early work demonstrated that aSyn can bind DNA in its supercoiled form (47) and that this binding can induce conformational changes both in the protein, increasing alpha-helical content (48), and in bound DNA, converting it into an altered B form (48, 49). aSyn can also directly bind a large subset of DNA promoter sequences (7), including the PGC-1a (42) and Notch (50) gene promoters, where it appears to often downregulate transcription. NMR spectroscopy demonstrates that the same N-terminal domain that binds curved, negatively charged phospholipid membranes mediates aSyn’s binding to the phosphate backbone of DNA (7). Single-molecule techniques have shown that aSyn binding can stretch DNA and increase its stiffness (51, 52), while fluorescence resonance energy transfer (FRET) assays developed to study noncanonical DNA structures show that aSyn stabilizes intermediate states on the pathway to DNA hairpin formation (53) and can bind to i-motifs (54). Although this work has started to reveal the mechanism of aSyn binding to DNA, important questions remain, including the role of potential disease-relevant aSyn posttranslational modifications in this process.

Phosphorylation of aSyn at serine-129 (pSyn) is a well-established marker of pathological forms of aSyn aggregates. While <4% of aSyn is phosphorylated at this site in normal brain, within Lewy bodies >90% of aSyn bears this posttranslational modification (55). In addition, human autopsy studies have shown that pSyn is the most sensitive immunohistochemical marker yet developed to detect Lewy body pathology (56). Although pSyn may be important for disease pathogenesis, and previous work suggests that aSyn phosphorylation can modulate its nuclear localization (7, 57, 58), little is known about how this phosphorylation may affect aSyn’s DNA-binding properties. Our previous work suggested that both aSyn and pSyn can bind DNA directly, but that phosphorylation can alter the nature of this interaction in potentially important ways (9). Given that a detailed understanding of how these two synuclein forms interact with DNA is still lacking, here we set out to test the properties of DNA that are important for aSyn binding and how serine-129 phosphorylation could modulate synuclein–DNA interactions.

## Results

### Alpha-synuclein and S129-phospho alpha-synuclein bind DNA in a DNA length-dependent manner

**Figure 1:**
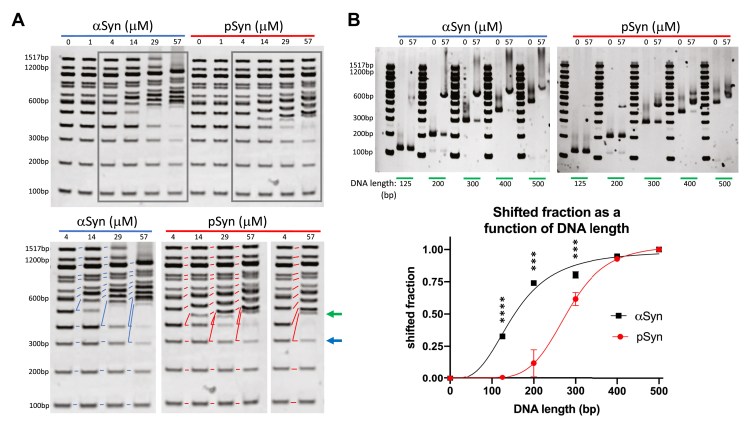
**Alpha-synuclein and phospho-synuclein bind DNA in a DNA length-dependent manner.***A*, *top*, EMSA with increasing alpha-synuclein (aSyn) and serine-129 phosphorylated alpha-synuclein (pSyn) concentrations with DNA of different lengths shows an upward shift of DNA bands greater than 200 bp at higher synuclein concentrations. Shifts produced by aSyn and pSyn of specific bands (within *gray rectangles*) are enlarged and shown below. *Bottom*, larger image of aSyn and pSyn EMSA shows upward shift of specific DNA bands longer than 200 bp depending on aSyn or pSyn concentration. For example, at 57 μM pSyn the 300 bp band exhibits both shifted (*green arrow*) and unshifted (*blue arrow*) species compared with the 4 μM concentration, where only an unshifted band (*blue arrow*) is present. *B*, *top*, shifts produced by aSyn (*left*) and pSyn (*right*) with individual DNA lengths (125, 200, 300, 400, and 500 bp). *Bottom*, group data show the fraction of different length DNAs shifted by 57 μM aSyn or pSyn. Both aSyn and pSyn binding to DNA depends on DNA length (aSyn EL50 = 153 bp, R^2^ = 0.986, deviation from zero slope *p* < 0.0001, Hill slope = 3.1; pSyn EL50 = 281 bp, R^2^ = 0.991, deviation from zero slope *p* < 0.0001, Hill slope = 6.0; four-parameter dose-response curve; N = 3 gels). aSyn produces more shift than pSyn of 125, 200 and 300 bp DNA (shifted fraction: aSyn-125 bp = 0.327 ± 0.015, pSyn = 125 bp = 0.007 ± 0.012, unpaired *t* test *p* < 0.0001; aSyn-200 bp = 0.740 ± 0.010, pSyn = 200 bp = 0.120 ± 0.104, unpaired *t* test *p* = 0.0005; aSyn-300 bp = 0.803 ± 0.025, pSyn = 300 bp = 0.617 ± 0.050, unpaired *t* test *p* = 0.0045; aSyn-400 bp = 0.947 ± 0.012, pSyn = 400 bp = 0.927 ± 0.015, unpaired *t* test *p* = 0.145; aSyn-500 bp = 1.000 ± 0.000, pSyn = 500 bp = 1.000 ± 0.000; N = 3 gels). Points at 0 DNA length set to 0.0 shifted fraction based on model for fitting purposes. EMSA, electrophoretic mobility shift assay.

Our previous work has shown that both aSyn and pSyn can bind 300 bp double-stranded DNA, but that aSyn produced multiple bound states, while pSyn produced only a single bound state under the conditions tested (9). To better understand this differential binding of aSyn and pSyn to DNA, we tested the ability of each synuclein to bind DNA of different lengths. We found that both aSyn and pSyn bind double-stranded DNA in a length-dependent manner (Fig. 1*A*). Increasing concentrations of synuclein incubated with a ladder containing DNA of various lengths and analyzed using a 10% polyacrylamide gel electrophoretic mobility shift assay (EMSA) system demonstrated that both proteins show little obvious interaction with DNA that is ≤200 bp in length, while longer DNAs are bound and shifted upward. Close examination of this shift due to synuclein binding revealed that at a given synuclein concentration, all DNA lengths above a certain critical length were bound and shifted upward, while below this critical length DNA was unbound, and there was no shift in these bands, and the DNA band in between these two lengths (at or near the critical length) exhibited both shifted (bound) and unshifted (unbound) bands (Fig. 1*A*). The value of these critical lengths, where the DNA exhibited both shifted and unshifted bands, decreased with increasing synuclein concentration. aSyn produced a range of bound states and, therefore, smearing of the signal at higher DNA lengths obscured the changes occurring to specific bands in the aSyn EMSA. This smearing did not occur with pSyn. This is consistent with our previous work showing that aSyn binding to 300 bp DNA produced a laddering effect due to the presence of multiple shifted bound states of different apparent lengths, while pSyn only produced a single shifted bound state (9). To understand the DNA length dependence of these band shifts better, without the complication of multiple-length DNA fragments in the same lane, we performed similar EMSAs using DNA of only a single length, ranging from 125 to 500 bp. These experiments also showed a clear length dependence to aSyn and pSyn DNA binding, with aSyn binding DNA better than pSyn at DNA lengths between 125 and 300 bp (Fig. 1*B*). The Hill slopes for binding of aSyn and pSyn were both positive, suggesting cooperative binding interactions between both synuclein forms and DNA, although we did not detect significant differences in the amount of cooperativity between aSyn or pSyn binding DNA. Given the relative simplicity of interpreting mobility changes with pSyn, due to the lack of smearing at high apparent DNA lengths, we also tested the ability of pSyn to shift ladder DNA under different gel conditions. We saw similar upward shifts of all DNA fragments above a certain critical length, no change below this critical length, and partial binding at or near these critical lengths when performing EMSAs with polyacrylamide gel concentrations ranging between 6 and 20% (Fig. S1*A*). To assay binding of aSyn to 300 bp DNA using another parallel technique to EMSA, we performed atomic force microscopy (AFM) on surfaces where DNA was applied either without aSyn or in the presence of 57 μM aSyn, a concentration where we measure robust aSyn-DNA binding in our EMSA experiments. These AFM data show evidence of increased apparent DNA thickness and a globular protein signal bound to 300 bp DNA in the condition where aSyn is present (Fig. S1*B*), suggesting that aSyn does bind 300 bp double-stranded DNA, as previous AFM studies have shown with longer DNA molecules (51).

The physiologically and pathologically relevant conformations of aSyn are subject to debate, and questions remain about the role/s of the unfolded monomeric state (59, 60), as well as a tetrameric structure that it can also form (61). Its ability to bind DNA resides in the N-terminal portion of the protein, and previous studies have noted the importance of electrostatic interactions between positively charged lysine residues in the N-terminal domain and the phosphate groups within the DNA backbone (7). This is the same N-terminal region that binds curved, negatively charged phospholipid membranes by adopting a discontinuous alpha-helical structure containing two antiparallel curved helices (38, 39, 40). The longest of these alpha-helices is made up of 48 amino acids (residues 45–92) and is measured to be less than 5 nm in length (40). In contrast, a 100 bp double-stranded DNA molecule is ∼34 nm, seven times longer than the DNA-binding domain of aSyn. These estimates and our data suggest that the length dependence of synuclein–DNA binding is not simply due to physical constraints preventing binding to shorter-length DNAs.

### Alpha-synuclein and S129-phospho alpha-synuclein bind DNA with blunt or 5′ overhanging ends with similar affinity

**Figure 2:**
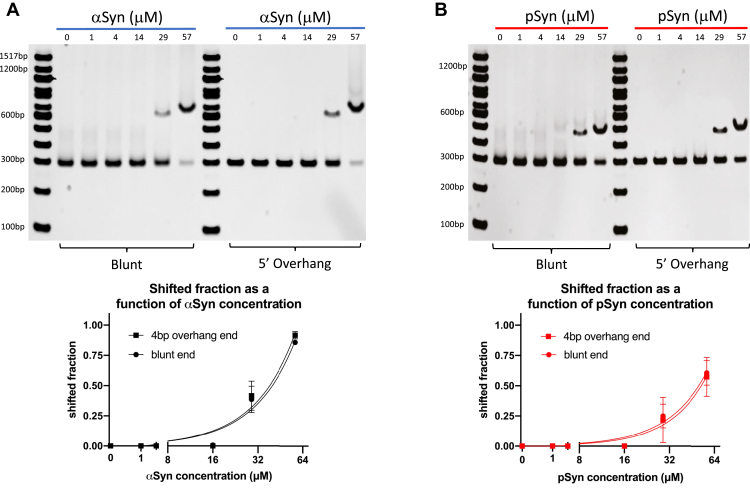
**Alpha-synuclein and phospho-synuclein bind DNA with blunt or 5′ overhanging ends with similar affinity.** Alpha-synuclein (aSyn, *A*) and serine-129 phosphorylated alpha-synuclein (pSyn, *B*) bind 300 bp DNA with blunt or 300 bp DNA with a 4 base 5′ overhanging end on both sides similarly (shifted fraction blunt end at 0–4, 16, 29, and 57 μM aSyn: 0.000 ± 0.000, 0.007 ± 0.012, 0.387 ± 0.110, 0.857 ± 0.006; shifted fraction overhanging ends at 0–14, 29 and 57 μM aSyn: 0.000 ± 0.000, 0.417 ± 0.121, 0.917 ± 0.031; comparison of shifted fraction of blunt and overhanging end DNA for each aSyn concentration by paired *t* test shows no significant differences, *p* between 0.059 and 0.423; variable slope dose-normalized response curve; N = 3 gels, x-axis aSyn concentration on log scale; shifted fraction blunt end at 0–14, 29 and 57 μM pSyn: 0.000 ± 0.000, 0.250 ± 0.098, 0.607 ± 0.101; shifted fraction overhanging ends at 0–14, 29 and 57 μM pSyn: 0.000 ± 0.000, 0.217 ± 0.188, 0.573 ± 0.161; comparison shifted fraction of blunt and overhanging end DNA for each pSyn concentration by paired *t* test shows no significant differences, *p* between 0.469 and 0.604; variable slope dose-normalized response curve; N = 3 gels, x-axis pSyn concentration on log scale).

We next tested the effect of DNA end structure on aSyn and pSyn binding. We used 300 bp DNA with either a blunt terminus on both ends or a 300 bp DNA with a 4 base 5′ overhang on each end. In our 10% polyacrylamide gel EMSA condition, both aSyn and pSyn bound these 300 bp DNAs similarly (Fig. 2). This suggests that the nature of the DNA end does not strongly influence aSyn and pSyn binding and that synuclein may not be a DNA end-binding protein, but rather a protein that binds DNA along its length, potentially in its major or minor groove. Similarly, changing the sequence of DNA, varying GC content from 56 to 67%, while keeping the length 300 bp, had no significant effect on the amount of or cooperativity of aSyn binding (Fig. S2*A*). Varying Tris concentration between 5 and 30 mM similarly also had no significant effects on the amount of or cooperativity of aSyn binding (Fig. S2*B*). These data suggest that synuclein is not recognizing very specific DNA sequences when binding, rather other aspects of the DNA molecule, such as stiffness or ability to adopt non-B forms, may be important. These more global DNA properties can be influenced by sequence, but our data suggest that aSyn and pSyn binding does not have a tight specificity for one particular DNA sequence. In our experiments, we only varied 300 bp DNA GC content between 56 and 67%, while previous work has shown that aSyn does bind 16 bp DNA sequences containing 100% GC content tightly and in the process can change them from B-form to an altered B-form of DNA (49). Our data are potentially consistent with this previous result (49), since only lower GC content sequences (56–67%) were tested, and it will be interesting in the future to explore the effects of wider ranges of GC content on synuclein–DNA binding. To test the importance of potential electrostatic interactions between aSyn and DNA in the binding reaction, the ionic strength of the composition of our EMSA buffer was increased. This was done by adding increasing amounts of the divalent cation Ca^2+^ to minimize the effect of osmolarity changes compared with a monovalent cation of comparable ionic strength. In these experiments, high concentrations of Ca^2+^, around 25 mM, did significantly decrease aSyn–DNA binding (Fig. S3). This potentially indicates the importance of electrostatic interactions between aSyn’s positively charged N-terminal domain and the negatively charged phosphate backbone of DNA for this binding.

### Alpha-synuclein and S129-phospho alpha-synuclein binding to DNA is facilitated by small molecules that bind in the minor groove but not the major groove

**Figure 3:**
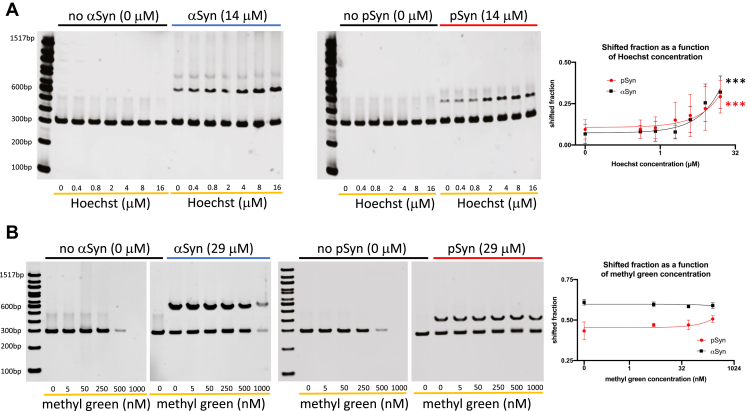
**Alpha-synuclein and phospho-synuclein DNA binding is increased by the minor groove-binding agent Hoechst 33258 and competes with the major groove-binding agent methyl green.***A*, increasing concentrations of the DNA minor groove binder Hoechst 33258 (0–16 μM) does not change the signal generated by DNA (*left side* of each gel) but does increase shifted fraction caused by 14 μM alpha-synuclein (aSyn) and serine-129 phosphorylated alpha-synuclein (pSyn; aSyn Hoechst EC50 = 21.29 μM, R^2^ = 0.653, deviation from zero slope *p* = 0.0003, three-parameter dose–response curve; N = 4 gels; pSyn Hoechst EC50 = 12.76 μM, R^2^ = 0.395, deviation from zero slope *p* = 0.0002, x-axis Hoechst concentration on log scale). *B*, in contrast, high concentrations (at 500 nM and above) of the DNA major groove binder methyl green decrease intercalation and/or fluorescence of the Sybr dye used to image DNA (*left side* of aSyn and pSyn gels). Addition of 29 μM aSyn or pSyn competes with methyl green and partially restores Sybr dye fluorescence signal. Methyl green does not affect the shifted fraction of DNA in the presence of 29 μM aSyn or pSyn at values where it does not reduce Sybr dye fluorescence (at 250 nM and less, aSyn: R^2^ = 0.205, deviation from zero slope *p* = 0.5473; pSyn: R^2^ = 0.748, deviation from zero slope *p* = 0.1351; N = 3 gels, x-axis methyl green concentration on log scale). The 14 and 29 μM aSyn and pSyn concentrations were chosen since these produced intermediate levels of shift, allowing for detection of possible changes with small-molecule dye addition.

To understand how aSyn and pSyn interact with double-stranded DNA, EMSAs under conditions where either the minor groove-binding small-molecule Hoechst 33258 or the major groove binder methyl green was present were performed. Samples were tested for possible interactions between Hoechst and the intercalating Sybr Gold fluorescent dye used to image DNA. Hoechst 33258 at concentrations from 0 to 16 μM had no appreciable effect on Sybr Gold fluorescence from 300 bp DNA (Fig. 3*A*). The addition of either aSyn or pSyn showed increased binding of the respective synuclein to DNA in a Hoechst 33258 dose-dependent manner, as evidenced by an increased fraction of shifted DNA (Fig. 3*A*). In contrast to the minor groove binder Hoechst 33258, the major groove-binding small-molecule methyl green reduced fluorescence from Sybr Gold at higher concentrations, with no appreciable effect between 0 and 25 nM, but a large reduction at 500 nM, and the abolition of Sybr Gold fluorescence at 1 μM (Fig. 3*B*). The addition of aSyn resulted in no increase in the fraction of shifted DNA at methyl green concentrations between 0 and 25 nM, indicating that methyl green does not promote aSyn-DNA binding (Fig. 3*B*). Between 250 and 500 nM, we did not quantify aSyn-DNA binding given the complication that methyl green reduces Sybr Gold fluorescence at these concentrations. However, it was observed that aSyn competes with methyl green and substantially restores Sybr Gold DNA fluorescence at these higher methyl green concentrations (Fig. 3*B*). Similar results were also seen with pSyn with evidence that pSyn was able to compete with methyl green even more effectively than aSyn to restore Sybr Gold DNA fluorescence (Fig. 3*B*).

These data suggest that the minor groove-binding agent Hoechst 33258 can alter the physical properties of DNA in a way that can promote aSyn and pSyn binding. Previous work has suggested that Hoeschst 33258, a bisbenzimide minor groove-binding molecule, can bend DNA upon binding (62, 63), potentially indicating that the ability of Hoechst 33258 to increase aSyn and pSyn DNA binding is related to a preference of synuclein for bent DNA conformations. In addition, the competitive interaction between the major groove binder methyl green (and not with the minor groove binder Hoechst 33258) with aSyn and pSyn suggests that synuclein itself is binding DNA within the major groove and not the minor groove. While the exact binding mechanism of Sybr Gold has not been extensively studied, this dye and other members of the class intercalate between base pairs (64). Our data suggest that methyl green (at higher concentrations) blocks Sybr Gold intercalation between base pairs, either by direct steric hindrance or by altering the parallel base-stacking interactions that are required for Sybr Gold intercalation. Increasing concentration of aSyn and pSyn appears to compete with methyl green binding to the major groove without altering Sybr Gold intercalation.

### Altering DNA concentration, while keeping alpha-synuclein concentration fixed, changes the nature of the bound complex, but this change does not happen with S129-phospho alpha-synuclein

**Figure 4:**
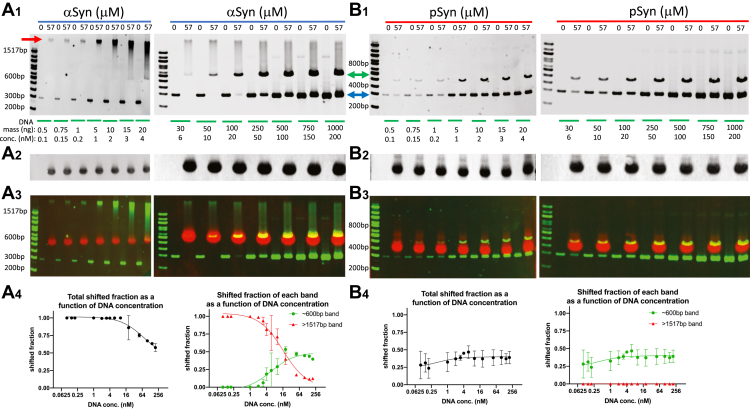
**Lowering DNA concentration shifts alpha-synuclein-bound complexes into a different state, but this does not happen with phospho-synuclein.***A*_1_, decreasing 300 bp DNA concentration from 200 nM to 0.1 nM with fixed alpha-synuclein (aSyn) concentration (57 μM) produces a change in the apparent length of the complex from to a lower value shift (running ∼600 bp, *green arrow*) to a higher value shift (running >1517 bp DNA, *red arrow*). Unshifted (unbound) DNA marked by the *blue arrow*. *A*_2_, Western blot showing aSyn protein loaded into each lane. *A*_3_, DNA gel (*green*) and aSyn Western blot (*red*) localization from the same experiment. *A*_4_, *left*, group data showing total shifted fraction as a function of DNA concentration (DNA IC50 = 72.24 nM, R^2^ = 0.919, three-parameter dose–response curve, N = 3 gels). *Right*, shifted fraction of high (>1517 bp) and low (∼600 bp) apparent length complexes (high length complex DNA IC50 = 16.26 nM, R^2^ = 0.961, three-parameter dose–response curve, N = 2 gels; low length complex DNA EC50 = 5.90 nM, R^2^ = 0.842, three-parameter dose–response curve, N = 2 gels). x-axis DNA concentrations on a log scale. *B*_1_, decreasing 300 bp DNA concentration from 200 nM to 0.1 nM with fixed serine-129 phosphorylated alpha-synuclein (pSyn) concentration (57 μM) produces no change in the apparent length of the bound complex (running ∼500 bp, *green arrow*). Unshifted (unbound) DNA marked by the *blue arrow*. *B*_2_, Western blot showing pSyn protein loaded into each lane. *B*_3_, DNA gel (*green*) and pSyn Western blot (*red*) localization from the same experiment. *B*_4_, *left*, group data showing little change in total shifted fraction as a function of DNA concentration (DNA EC50 = 0.38 nM, R^2^ = 0.116, three-parameter dose–response curve, N = 3–4 gels). *Right*, shifted fraction of each complex shows no detectable high (>1517 bp), and only detectable low (∼500 bp) apparent length complexes. x-axis DNA concentrations on a log scale.

To understand the differing nature of the bound state/s between aSyn or pSyn with DNA, we performed a set of experiments where aSyn concentration was fixed at 57 μM and the DNA concentration was varied between 0.1 and 200 nM. At higher DNA concentrations (20–200 nM), most DNA signals were found in either the unbound band running at its true 300 bp length or in a single discrete shifted band, indicating a single bound state running at an apparent length of ∼600 bp (Fig. 4*A*). At intermediate DNA concentrations (2–10 nM), the unbound band was not detectable, and all signal was either in the discrete band at ∼600 bp or in a smear of higher apparent lengths (Fig. 4*A*). At the lowest DNA concentrations (0.1–1 nM), all the signals appeared in a single, discrete high apparent length band running at >1517 bp (Fig. 4*A*). In contrast to the aSyn data, with pSyn there was no change in the pattern of binding at any tested DNA concentration (0.1–200 nM). At all concentrations, ∼60% of the DNA signal was in a single, discrete unshifted (unbound) band running at 300 bp, and the other ∼40% was present in a single shifted (bound) band running at ∼500 bp (Fig. 4*B*).

### Altering protein concentration, while keeping DNA concentration fixed, changes the nature of the bound complex with alpha-synuclein, but this does not happen with S129-phospho alpha-synuclein

**Figure 5:**
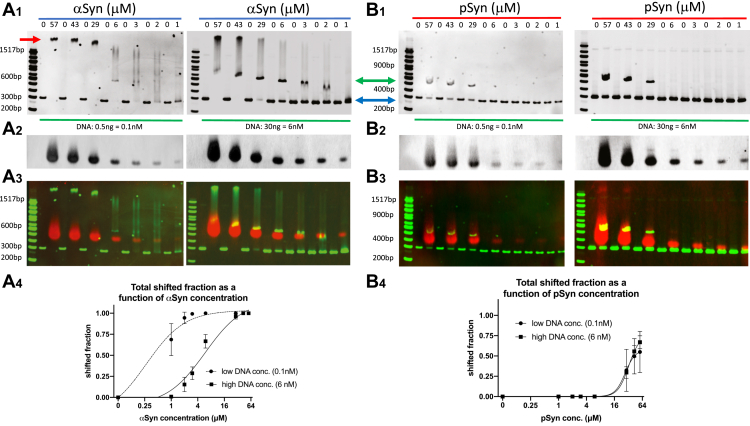
**Increasing alpha-synuclein concentration shifts alpha-synuclein-bound complexes into a different state, but this does not happen with phospho-synuclein.***A*_1_, decreasing alpha-synuclein (aSyn) protein concentration from 57 to 1 μM with 300 bp DNA concentration fixed at either 0.1 nM (*left*) or 6 nM (*right*) produces a change in the apparent length of the complex from a higher value shift (running >1517 bp DNA, *red arrow*) to a lower value shift (running ∼600 bp, *green arrow*). Unshifted (unbound) DNA marked by the *blue arrow*. *A*_2_, Western blot showing aSyn protein loaded into each lane. *A*_3_, DNA gel (*green*) and aSyn Western blot protein (*red*) localization from the same experiment. *A*_4_, group data showing shifted fraction as a function of aSyn concentration for the two different fixed DNA concentrations. The low DNA concentration curve is *left*-shifted (aSyn EC50: low DNA conc. = 0.273 μM, R^2^ = 0.619; high DNA conc.=5.846 μM, R^2^ = 0.977, three-parameter dose–response curve; N = 3–4 gels, x-axis aSyn concentration on log scale). *B*_1_, decreasing serine-129 phosphorylated alpha-synuclein (pSyn) protein concentration from 57 to 1 μM with 300 bp DNA concentration fixed at either 0.1 nM (*left*) or 6 nM (*right*) produces only a complex with an apparent length of ∼500 bp (*green arrow*) at the higher pSyn concentrations. Unshifted (unbound) DNA marked by the *blue arrow*. *B*_2_, Western blot showing pSyn protein loaded into each lane. *B*_3_, DNA gel (*green*) and pSyn Western blot (*red*) localization from the same experiment. *B*_4_, group data showing a similar shifted fraction as a function of pSyn concentration for the two different fixed DNA concentrations (pSyn EC50: low DNA conc.=27.34 μM, R^2^ = 0.779; high DNA conc.=32.49 μM, R^2^ = 0.981, four-parameter dose–response curve; N = 3 gels, x-axis pSyn concentration on log scale).

We next performed a set of experiments where synuclein protein concentration was varied between 0 and 57 μM, while DNA concentration was fixed at either a relatively low (0.1 nM) or intermediate (6 nM) concentration. In these experiments, aSyn-DNA binding once again exhibited a range of bound states limited on the lower end by a discrete state running at ∼600 bp, favored at the higher DNA concentration (6 nM) when aSyn concentration was relatively low (2–29 μM)—a low aSyn-to-DNA ratio (Fig. 5*A*). While at a high aSyn-to-DNA ratio, when DNA was at the lower concentration (0.1 nM) and aSyn was relatively high (43–57 μM), a single larger discrete state was favored, running at >1517 bp (Fig. 5*A*). At intermediate aSyn-to-DNA ratios, a range of bound states between these two limits was observed (Fig. 5*A*). Once again, this range of states was not observed with pSyn, and conditions varying pSyn produced only a single bound state running at ∼500 bp at high pSyn concentrations (29–57 μM, Fig. 5*B*).

### Only alpha-synuclein, and not S129-phospho alpha-synuclein, binds circular DNA

Our results varying either DNA or protein concentration, while keeping the other fixed (Figs. 4 and 5), are consistent with a model where aSyn and pSyn are both able to bind DNA in at least one state, which shifts the mobility of the bound complex to run as a discrete band at an apparent length of ∼500 to 600 bp. Over the DNA concentration range tested (0.1–200 nM) for both synuclein proteins, this single bound state is the only complex formed with pSyn. aSyn, however, produces multiple bound states. This range of aSyn-DNA complexes occurs, however, over a specific interval when using 300 bp DNA. High DNA concentrations (20–200 nM) favor a single discrete state that runs at an apparent length of ∼600 bp, while low DNA concentrations (0.1–1 nM) favor a much higher, but still single discrete state, at an apparent length >1517 bp. At intermediate DNA concentrations (2–10 nM), a variety of aSyn-DNA bound states are present that span this range (∼600 to >1517 bp). These data suggest a model where pSyn can bind DNA as a single monomer (or potentially small multimer) to the DNA molecule, but that only one such binding event occurs per DNA molecule. In contrast, aSyn can make a similar one-synuclein-to-one DNA molecular complex and that is favored at relatively high DNA concentrations, but when DNA concentrations are relatively low, multiple monomers (or small multimers) bind as discrete events along the DNA length. Consistent with this, there is a saturation of aSyn-DNA binding, presumably when all possible sites on a single molecule of DNA are occupied to create the largest possible bound complex (running at >1517 bp). Our results are similar to previous work using atomic force microscopy to image aSyn binding to linear 1000 bp DNA molecules, where at lower aSyn concentration (0.1 μM), aSyn molecules bind at discrete sites randomly distributed up and down the DNA molecule. At a higher aSyn concentration (40 μM), however, the entire DNA molecule was evenly coated with a single layer of aSyn (51). In our experiments, at high aSyn-to-DNA ratios, the data are also consistent with an increasing number of aSyn molecules binding one DNA molecule up to a saturation point. aSyn binding significantly changes the conformation of DNA so that it runs at a much higher apparent length (>1517 bp). This suggests that aSyn may be bending DNA into specific shapes, potentially circle-like forms, which are known to run much more slowly through polyacrylamide gels (65). This kind of bending reaction upon binding is inhibited by phosphorylation at serine-129 in pSyn, since even at high pSyn-to-DNA ratios pSyn binds DNA in a one-to-one fashion that does not further slow its migration through the polyacrylamide gel.

**Figure 6:**
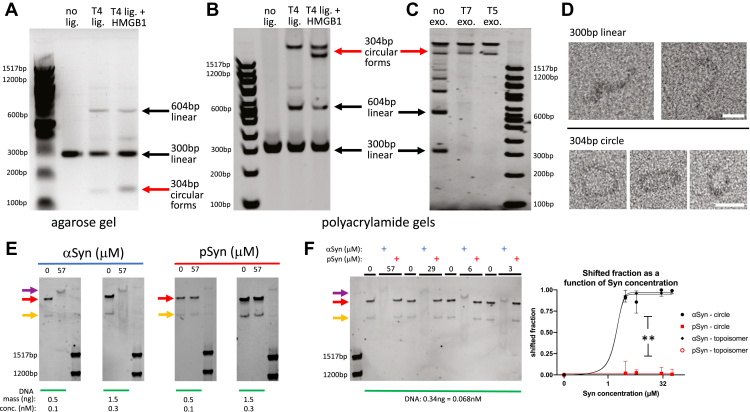
**Alpha-synuclein binds 304 bp circular DNA forms and phospho-synuclein does not.***A*, agarose gel electrophoresis shows 300 bp linear DNA (with additional 4 base 5′ overhanging ends on each side) treated with no T4 ligase, T4 ligase, or T4 ligase and the DNA bending protein HMGB1. The untreated sample only contains 300 bp linear DNA. The T4 ligase treated sample contains 300 bp and 604 bp linear DNA and 304 bp circular forms (which run faster than their linear counterpart in the agarose gel system). The sample treated with T4 ligase and HMGB1 shows increased formation of 304 bp circular DNA forms. *B*, polyacrylamide (6%) gel electrophoresis shows 300 bp linear DNA treated with no T4 ligase, T4 ligase, or T4 ligase and the DNA bending protein HMGB1. The untreated sample only contains 300 bp linear DNA. The T4 ligase treated sample contains 300 bp & 604 bp linear DNA and 300 bp circular forms (which run slower than their linear counterpart in the polyacrylamide gel system). The sample treated with T4 ligase and HMGB1 shows the formation of a new 304 bp circular DNA topoisomer form (which runs somewhat faster due to its more compact nature). *C*, 300 bp linear DNA first treated with T4 ligase and HMGB1 (to produce some circular forms, as in *B*), then with either T7 or T5 exonuclease to remove linear DNA molecules, shows the expected resistance to exonuclease treatment of the 304 bp circular DNA forms. *D*, *top*, negative stain transmission electron microscopy of 300 bp linear DNA without ligase treatment shows linear molecules of the expected length (∼100 nm). Scale bar 25 nm. *Bottom*, linear DNA treated with T4 ligase shows 304 bp circular molecules with the expected diameter (∼30 nm). Scale bar 25 nm. *E*, only aSyn, and not pSyn, causes a shift of the 304 bp circular DNA forms created in the presence of HMGB1 (*red arrows*: circular form, *orange arrows*: circular topoisomer) to a higher apparent length (*purple arrow*). *F*, *left*, synuclein concentration dependence of shift of circular DNA forms created in the presence of HMGB1 shows that only aSyn causes a shift at these concentrations and pSyn does not. *Right*, group data showing shifted fraction of 304 bp DNA circles and circular topoisomers as a function of synuclein concentration (aSyn: EC50 circular DNA = 1.608 μM, R^2^ = 0.967; EC50 circular DNA topoisomer = 1.637 μM, R^2^ = 0.993; pSyn: EC50 circular & circular DNA topoisomer undefined; at 3–57 μM one-way ANOVA for each concentration, *p* between <0.0001 and 0.0079; post-hoc Tukey tests shows no significant differences between two [circle *versus* topoisomer] aSyn conditions *p* between 0.453 and 0.960, and two [circle *versus* topoisomer] pSyn conditions *p* = 0.252–0.956; there are significant differences between two [aSyn *versus* pSyn] circle conditions *p* = 0.001–0.028, and the two [aSyn *versus* pSyn] topoisomer conditions *p* ≤ 0.0001–0.002; three-parameter dose–response curve, N = 3 gels, x-axis synuclein concentrations on log scale).

Given our data suggesting that synuclein proteins promote DNA bending upon binding (Fig. 3) and that multiple aSyn molecules may bend a single DNA molecule into a circle-like form (Figs. 4 and 5), we next tested whether synuclein proteins bind to circular DNA molecules that are forced by their topology into a bent conformation. We converted the linear 300 bp DNA (with four base overhangs on each end) used in our previous experiments into a 304 bp circular form using T4 ligase, both in the presence and absence of the DNA bending protein HMGB1 (Fig. 6, *A* and *B*). To remove any remaining linear DNA and confirm their circular state, we incubated this product with either T5 or T7 exonuclease. These data demonstrate the formation of 304 bp circular DNA, including an additional specific topoisomer that was only present when circular DNA, was formed in the presence of HMGB1 (Fig. 6, *B* and *C*). To further check the circular nature of these DNA, we performed negative stain transmission electron microscopy on the linear and circular DNA samples. These data show the expected presence of linear and circular forms in their respective samples (Fig. 6*D*). These circular forms run in a polyacrylamide gel system at a high apparent length (>1517 bp), consistent with previous work showing that circular DNA is much less mobile than its linear counterpart in polyacrylamide gels and that circular topoisomers are more compact and therefore migrate somewhat faster than topologically simple circles (65). Interestingly, incubation of these 304 bp circular forms with aSyn produced clear evidence of binding, with a shift of both the circular and circular topoisomer DNA bands (Fig. 6, *E* and *F*), and shift of the simple circular DNA form created without the presence of HMGB1 (Fig. S4). pSyn was unable to bind either of these circular forms and produced no appreciable shift (Figs. 6, *E* and *F* and S4). These data suggest that the high apparent length complex formed by aSyn binding to linear DNA under conditions that favor this bound state (high aSyn-to-DNA ratio, Figs. 4*A* and 5*A*) may be similar in conformation to circular DNA bound to aSyn, since their bound complexes share similar properties, including apparent length, and binding dependence on aSyn concentration (Fig. 6*F*).

### Mutant alpha-synuclein and beta- and gamma-synuclein do not bind linear DNA similarly to wild-type alpha-synuclein

**Figure 7:**
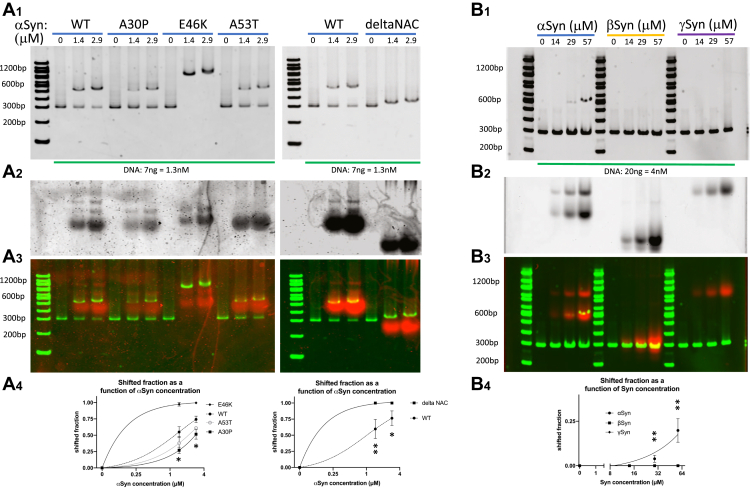
**Mutant alpha-synuclein and beta- and gamma-synuclein do not bind DNA like wild-type alpha-synuclein.***A*_1_, *left*, increasing concentrations of wild-type (WT) alpha-synuclein (aSyn) and disease-causing point mutations (A30P, E46K, A53T) shift 300 bp DNA to higher apparent lengths, with A30P and A53T aSyn being less efficient at shifting than WT, and E46K being more efficient and shifting to a higher apparent length than WT. *Right*, deletion of the central aggregation-prone NAC domain of aSyn (deltaNAC) increases the efficiency of shifting but reduces the apparent length of the shifted species compared to WT. *A*_2_, Coomassie stain showing aSyn proteins loaded into each lane. *A*_3_, DNA gel (*green*) and Coomassie stain (*red*) localization from the same experiment. *A*_4_, group data showing shifted fraction as a function of aSyn concentration (aSyn R2: WT = 0.980, A30P = 0.963, E46K = 0.999, A53T = 0.965; shifted fraction 1.4 μM aSyn: WT = 0.550 ± 0.082, A30P = 0.270 ± 0.047, E46K = 0.975 ± 0.028, A53T = 0.379 ± 0.102; shifted fraction 2.9 μM aSyn: WT = 0.743 ± 0.049, A30P = 0.515 ± 0.074, E46K = 0.996 ± 0.007, A53T = 0.611 ± 0.010; at 1.4 μM one-way ANOVA *p* < 0.0001; post-hoc Tukey tests WT *versus* A30P, E46K, A53T all *p* between 0.0002 and 0.0449; at 2.9 μM one-way ANOVA *p* < 0.0001; post-hoc Tukey tests WT *versus* A30P, E46K, A53T all *p* between 0.0004 and 0.0174; shifted fraction 1.4 μM aSyn: WT = 0.597 ± 0.148, deltaNAC = 1.000 ± 0.000; shifted fraction 2.9 μM aSyn: WT = 0.764 ± 0.113, deltaNAC = 1.000 ± 0.000; at 1.4 μM *t* test *p* = 0.0092; at 2.9 μM *t* test *p* = 0.0225; three-parameter dose–response curve; N = 3 gels, x-axis aSyn concentrations on log scale). *B*_1_, increasing aSyn concentration produces a shift of 300 bp DNA to an apparent length of ∼600 bp, while beta-synuclein (bSyn) and gamma-synuclein (gSyn) produce no such shift. *B*_2_, Coomassie stain showing synuclein proteins loaded into each lane. *B*_3_, DNA gel (*green*) and Coomassie stain (*red*) localization from the same experiment. *B*_4_, group data showing shifted fraction as a function of synuclein concentration (aSyn R2 = 0.791; shifted fraction: 29 μM aSyn = 0.039 ± 0.020, 57 μM aSyn = 0.199 ± 0.066, all other values including for all bSyn and gSyn concentrations produced an undetectable shift = 0.0; at 29 μM one-way ANOVA *p* = 0.0087; post-hoc Tukey test aSyn *versus* bSyn, and aSyn *versus* gSyn *p* = 0.0139; at 57 μM one-way ANOVA *p* = 0.0010; post-hoc Tukey test aSyn *versus* bSyn, and aSyn *versus* gSyn *p* = 0.0017; three-parameter dose–response curve; N = 3 gels, x-axis Syn concentrations on log scale).

To determine the potential effects of mutations in aSyn on 300 bp linear DNA binding, we tested under similar conditions, three disease-causing point mutations (A30P, E46K, A53T) and a deletion form where the central nonamyloid beta-component (NAC) domain, which is important for fibrillar aggregation of the protein, was removed (delta NAC). We tested all proteins at a low DNA concentration (1.3 nM), which shows moderate levels of binding when aSyn is used (Fig. 7*A*). All mutant forms of aSyn were capable of binding and shifting DNA, although with different efficiencies and to different apparent lengths. Two of the disease-causing point mutations (A30P, A53T) were less efficient at binding DNA than wild-type (WT) but did shift DNA to a similar apparent length, while the other disease-causing point mutation tested (E46K) was more efficient at binding DNA and shifted it to a higher apparent length (Fig. 7*A*). The delta NAC form was also more efficient at binding DNA but shifted it to a lower apparent length than WT (Fig. 7*A*).

To test the ability of the two aSyn paralogs, beta- and gamma-synuclein (bSyn and gSyn, respectively), to bind DNA we next tested all three synuclein proteins under similar conditions. We tested all three proteins at an intermediate DNA concentration (4 nM), which shows clear binding when aSyn is used (Fig. 7*B*). Unlike aSyn, however, we did not detect appreciable binding of either bSyn or gSyn to DNA (Fig. 7*B*). We next wanted to test the effect of lowering DNA or bSyn concentration on potential binding between these two molecules. Interestingly, lowering DNA concentration (0.2–4 nM) while keeping bSyn concentration fixed (57 μM) showed no obvious change at the higher DNA concentrations, but at the lower DNA concentrations, the DNA signal was reduced so that at the lowest DNA concentration (0.2 nM, highest bSyn-to-DNA ratio), no DNA signal could be detected at all (Fig. S5*A*). A similar phenomenon was seen when DNA concentration was fixed at 0.2 nM and bSyn concentration varied (3–57 μM), where at the highest bSyn-to-DNA ratio, DNA signal was absent (Fig. S5*B*). A similar set of results was also obtained when testing the ability of gSyn to bind DNA (Fig. S6), where the highest gSyn-to-DNA ratios produced a loss of DNA signal. The physical interpretation of this loss in DNA signal when either bSyn or gSyn is present at a high Syn-to-DNA ratio is not clear. There is no evidence that this is due to either DNA trapped in the well or DNA migrating at a much faster speed under these conditions (data not shown). This suggests that bSyn and gSyn may be changing the conformation of DNA in a way that prevents the Sybr Gold fluorescence used to detect DNA, potentially by directly inhibiting Sybr Gold intercalation into B form DNA or by altering the DNA from a B form to another non-B form that Sybr Gold does not bind, as has been previously suggested to occur when synuclein binds DNA (48, 49). Another possibility is that bSyn and gSyn could be degrading DNA *via* a nuclease function. It has been shown that oxidized aSyn species can act as nucleases (66), although in our experiments we do not detect evidence of the DNA degradation that would be expected in this case and have also chelated the divalent metal ions required for many nucleases to function.

## Discussion

Our data show that both aSyn and pSyn can bind DNA, but do so in a DNA length-dependent manner. DNA molecules shorter than ∼300 bp are not significantly bound under the conditions tested, while DNA longer than this is bound and shifted in our EMSA assay. Interestingly, DNA that is ∼300 bp long is often only partially bound, producing discrete unshifted (unbound) and shifted (bound) bands. DNA sequence or end structure does not strongly modulate this binding, arguing that another length-dependent physical property of DNA is important for binding. One potential possibility is DNA stiffness. Previous work has shown that DNA >300 bp in length is flexible enough to easily bend into a circular form, while DNA <200 bp does not easily form circles without the use of specific DNA bending proteins because of its inherent stiffness (67). DNA that is ∼200 to 300 bp long is at a length (and stiffness) where unaided circle formation starts to be possible. This suggests that the interaction of aSyn and pSyn with DNA may be modulated by DNA stiffness in such a way that binding requires a structural change in DNA that cannot occur when it is too stiff. Such a structural change could involve DNA bending and our data using the bisbenzimide DNA dye Hoechst 33258, a dye known to bend DNA (62, 63), suggests that DNA bending promotes aSyn and pSyn binding. aSyn is able at high aSyn-to-DNA ratios to produce a structurally different complex than at low aSyn-to-DNA ratios. Our data suggest that this complex may involve multiple aSyn monomers or multimers binding DNA until all possible sites are occupied. The fully saturated complex runs at a much higher apparent length and may involve DNA bending into a circle-like state. Serine-129 phosphorylation strongly inhibits the formation of this potential circle-like form, and our experiments testing aSyn and pSyn binding directly to 304 bp circular DNA are consistent with this interpretation. bSyn and gSyn do not bind DNA similarly to aSyn under the conditions we have used. These data suggest that aSyn has unique properties among the synuclein family members in terms of its ability to bind DNA and stabilize circle-like forms. It also suggests that serine-129 phosphorylation is a potential regulatory switch that can inhibit this process. Interestingly, known disease-relevant point mutations in alpha-synuclein modulate DNA binding in complicated ways, with A30P and A53T reducing binding and E46K increasing binding. Removal of the NAC domain also increases DNA binding, but produces a state with a lower apparent length.

**Figure 8:**
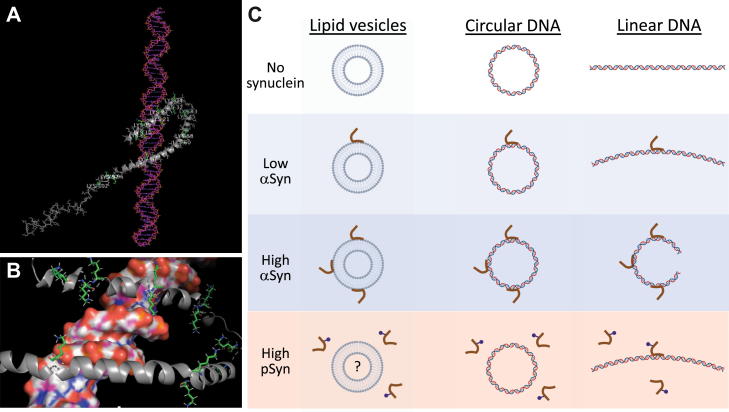
**Model of alpha-synuclein binding to double-stranded DNA and the potential similarities between this DNA binding and binding to phospholipid vesicles.***A*, single image from an animation (see Fig. S7 for full animation) showing a model of aSyn bound to DNA. The aSyn coordinates come from previous work showing its structure bound to a phospholipid micelle (40). The two curved alpha-helices of the N-terminal domain of aSyn fit into consecutive major grooves of B form DNA. Lysine residues within the protein are labeled. The C-terminal domain, containing the serine-129 phosphorylation site, is poorly resolved in previous structural work, so it is not clear how this phosphorylation may modulate DNA binding. *B*, single image from an animation (see Fig. S8 for full animation) showing a model of aSyn bound to DNA with 12 lysine residues labeled, using the same structural coordinates as in (*A*). In this particular model, potential electrostatic interactions between lysine-21, -23, and -80 with the phosphate backbone of DNA are present. *C*, *left*, ∼30 nm (in diameter) phospholipid vesicle shown in the presence of no synuclein, low or high alpha-synuclein (aSyn), and high serine-129 phosphorylated alpha-synuclein (pSyn). At low aSyn concentrations, aSyn binds the vesicle given its relatively high affinity for these structures. At high aSyn concentrations, multiple aSyn molecules are bound. To our knowledge, the effects of serine-129 phosphorylation on the interaction of aSyn with phospholipid bilayer vesicles with a diameter of ∼30 nm have not yet been tested. We speculate that serine-129 phosphorylation may inhibit binding in this context. *Middle*, 300 bp circular DNA having a diameter of ∼30 nm. At low concentrations, aSyn binds DNA given its high affinity for the curved negatively charged DNA surface (due to the phosphate backbone). At high aSyn concentrations, multiple aSyn molecules are bound, analogous to their binding to phospholipid vesicle membranes of similar geometry (*left*). Serine-129 phosphorylation inhibits the ability of synuclein to bind 300 bp circular DNA. *Right*, 300 bp linear DNA having a length of ∼100 nm. At low aSyn concentrations, aSyn binds as a monomer and bends linear DNA. At high aSyn concentrations, multiple aSyn molecules bind linear DNA and bend it into a conformation that resembles a DNA circle (*middle*). At high pSyn concentrations, pSyn binds as a monomer and bends linear DNA, but is not able to bend it into a circle-like form.

It strikes us that 200 to 300 bp DNA when bent into a circle has a diameter that is ∼20 to 30 nm. This is similar to the diameter of a synaptic vesicle found at the nerve terminal to which aSyn is known to bind (1, 2, 3). Several studies have shown that aSyn binds synaptic vesicles through its N-terminal domain, which can form two concave-curved antiparallel alpha-helices that associate with the convex-curved outer leaflet of the vesicle lipid bilayer (38, 39, 40). This highly curved phospholipid bilayer of a ∼20 to 40 nm (in diameter) synaptic vesicle is thought to present a geometry that strongly favors aSyn binding, compared with other membranous compartments found in the terminal that have less curved geometries. The concave-curved antiparallel alpha-helices in aSyn’s N-terminus are amphipathic, favoring strong interactions between their hydrophobic face and the lipid environment within the membrane and between the 12 positively charged lysine residues found on the opposite face (between amino acids 1–80) and the membrane’s negatively charged phosphate head groups. Previous work using NMR spectroscopy has shown that aSyn binds DNA by interacting with the phosphate backbone, using the same N-terminal region that binds curved phospholipid membranes (7). Given the comparable geometries of a 200 to 300 bp long DNA molecule bent into a circle and a synaptic vesicle, this suggests to us that aSyn may have evolved to bind negatively charged convex surfaces of discrete geometries using its N-terminal domain and does so with both synaptic vesicles and DNA in a similar fashion. Using structural data taken from aSyn bound to micelles (composed of 70:30 mixtures of dodecyl phosphocholine:sodium dodecylsulfate) (40) to make a docking model with double-stranded DNA suggests that each N-terminal antiparallel alpha-helix can fit within a major groove so that two consecutive major grooves of DNA are occupied. This model positions aSyn’s hydrophobic helix face close to individual DNA bases and also positions several helix lysine residues within proximity of the DNA phosphate backbone, allowing for possible electrostatic interactions with the DNA phosphate backbone (Fig. 8, *A* and *B*, S7 and S8). At the presynaptic terminal, aSyn’s binding to negatively charged, curved phospholipid surfaces is important for modulating synaptic vesicle cycling and neurotransmission (30, 31, 32, 33, 34), while in the nucleus it could be important for modulating DNA structure-dependent processes where aSyn has been implicated, such as transcription (42, 43) and DSB repair (9). It will be important in the future to test this model in both DNA and spherical phospholipid bilayer systems that present negatively charged curved surfaces of the appropriate geometry to determine the details of alpha-synuclein binding and whether similarities between its binding to DNA and curved phospholipid surfaces exist.

Several studies have tested the effects of serine-129 phosphorylation on aSyn’s membrane-binding properties. In the context of binding synaptosomal membranes (a mixture of the presynaptic vesicle, organelle, and plasma membranes), serine-129 phosphorylation did not affect the binding of the WT aSyn protein, but did increase the membrane binding of the disease-associated A30P mutation (which has reduced membrane binding compared with WT at baseline) (68). Work in a micelle model system produced similar results: that serine-129 phosphorylation did not affect lipid-bound aSyn, but could disrupt long-range N- and C-terminal interactions within the protein when it is not lipid-bound (69). This argues that long-range N- and C-terminal interactions in the protein occur and can be modulated by phosphorylation. In contrast, other work suggests that serine-129 phosphorylation does inhibit binding to certain membranes, such as ER, Golgi, and mitochondrial membranes in neurons (70) or plasma membranes in yeast (71). In planar lipid nanodisc (72) and unilamellar vesicle (73) systems, the alpha-synuclein C-terminus was disordered even without serine-129 being phosphorylated. Unfortunately, to our knowledge, the potential effects of serine-129 phosphorylation on isolated presynaptic vesicles, or model spherical phospholipid bilayer systems with similar chemical composition and ∼20 to 40 nm (in diameter) geometry, have not been tested to date. Our data showing that aSyn binds 304 bp circular DNA, which has a similar geometry to ∼30 nm synaptic vesicles, while pSyn does not, lead us to propose a model where high concentrations of aSyn favor the formation of circle-like DNA structures (Fig. 8*C*). We also speculate, based on potential analogies between DNA circle and phospholipid vesicle binding, that serine-129 phosphorylation may reduce aSyn binding to presynaptic vesicles, as it does for circular DNA (Fig. 8*C*). It is possible in disease, where aSyn’s ability to properly bind synaptic vesicles could be compromised (35), that a similar deficiency in properly binding and bending DNA also occurs and leads to detrimental cellular consequences due to the dysregulation of nuclear processes that require these synuclein–DNA interactions. It will be interesting in future work to directly test the validity of our proposed model by using other techniques that are complementary to the primarily EMSA approaches we have used here. For example, examining the high-resolution structure of aSyn bound to linear and circular DNA forms, potentially using X-ray diffraction or rapidly advancing cryo-EM approaches, could allow direct visualization of the synuclein–DNA complex. Also, since EMSA is a biochemical technique requiring purified components, it cannot directly measure synuclein–DNA interactions within the cellular milieu. It will also be important in the future to study these interactions using other biophysical and cell biological approaches that can assay and measure the potential functional consequences of aSyn binding and bending nuclear DNA.

Previous work has also suggested that phosphorylation of aSyn by Polo-like kinases (PLK) can play important roles in regulating the amount present in the nucleus, and that the PLK family member PLK2 specifically phosphorylates serine-129 in the soluble nuclear fraction (74). This effect is likely complicated, however, since either overexpressing PLK2 or PLK3 (57) or genetically deleting PLK2 (75) produces data consistent with alpha-synuclein phosphorylation at serine-129 by these kinases as promoting export of synuclein from the nucleus and into the cytoplasm. In contrast, other work suggests that when synuclein is forced into the nucleus by fusing it with a nuclear localization sequence and then mutated at serine-129 to the unphosphorylatable residue alanine, accumulation in the cytoplasm is promoted (7). Further work is required to understand the details of synuclein shuttling between the nuclear and cytoplasmic compartments, but these data suggest that the identity of the kinase phosphorylating serine-129 or other cophosphorylation events on other residues of the protein could be important for the direction in which it travels (into or out of the nucleus).

Our data suggest a new framework for understanding the role of aSyn in the nucleus and its biophysical interactions with the DNA located there. The ability of aSyn to bend DNA into distinct circle-like conformations may play a role in how it inhibits transcription from specific promoters (7, 42, 50) and/or facilitates forms of DSB repair (9). Our work also highlights the important role of serine-129 phosphorylation in regulating this process, acting as a potential switch that causes aSyn to unbind from DNA, and therefore allowing the DNA to relax from a circle-like conformation into a more linear state. Further exploration of these processes could help elucidate the poorly understood functions of nuclear aSyn and how they may be relevant to important forms of neurodegeneration and cancer.

## Experimental procedures

### Reagents

EMSA reaction buffers and protein storage buffers were formulated using either MilliQ purified H_2_O or filtered molecular biology grade H_2_O (0.1 μm filter). pH was determined using a pH meter and adjusted with 1 M HCl or 5 M NaOH before vacuum filtration (0.2 μm filter). Electrophoresis buffers were made with MilliQ purified H_2_O and 10× TBE or 50× TAE stock solutions.

### DNA generation

300 bp linear and 304 bp circular double-stranded DNAs were created using the 300 bp band from a 100 bp ladder (New England Biolabs, cat. # N3231) as the template DNA. After each extraction and synthesis step described, constructs were cleaned using the QIAquick PCR clean-up kit (Qiagen, cat. # 28104) according to the manufacturer’s protocol. An extra wash step and two extra spin steps were added before elution of DNA into buffer in the Qiagen protocol to reduce ethanol contamination. The final products were eluted into H_2_O, 1× TE or Buffer EB (Qiagen; 10 mM Tris, pH 8.5) and stored at −20 °C until use. The concentration and purity of DNA were determined by obtaining 260/280 nm and 260/230 nm readings with a Nanodrop spectrophotometer. 125, 200, 300, 400, and 500 bp linear DNA used for testing length dependence and 300 bp DNA with varying GC content were synthesized using gBlocks (IDT).

#### Gel extraction

100 bp DNA ladder (New England Biolabs, cat. # N3231) in 1× Orange Loading Dye (Li-Cor, cat. # 927-10100) was separated on a 1.5% TAE agarose gel in 1× TAE at 175 V (2 h at room temperature, RT) in the presence of 1× Sybr Safe (Invitrogen, cat. # S33102) using the QIAquick Gel Extraction Kit (Qiagen, cat. # 28704) using the manufacturer’s recommended protocol, with the addition of two extra wash and spin steps to minimize ethanol contamination before final elution.

#### Polymerase chain reaction

Between 10 and 100 ng of 300 bp template DNA was added to 1 μM of forward and reverse primers (IDT, forward: 5′ TCGAGCAGGCAGAACGG 3′, reverse: 5′ TGCCTGGTAGGCGTCC 3′) using 1× Phusion HF PCR Master Mix with HF Buffer (New England Biolabs, cat. # M0531). 300 bp linear dsDNA with additional 4 base 5′ overhangs on each end were generated from our 300 bp template DNA using PCR primers (IDT, forward: 5′ TTAATGAATTCGAGCAGGCAGAAC 3′, reverse: 5′ AATTAGAATTCTGCCTGGTAGGCGT 3′) that created a 320 bp dsDNA with EcoRI restriction enzyme sites on each end. When cut with EcoRI (1–16 h at 37 °C, EcoRI-HF, New England Biolabs, cat. # R3101), the 300 bp linear dsDNA with the 4 base 5′ overhanging ends was produced and the reaction stopped with Proteinase K treatment (1 h at 37 °C, New England Biolabs, cat. # P8111). All DNA was cleaned using the QIAquick PCR clean-up kit (Qiagen, cat. # 28104) before use.

#### Circular DNA preparation

Cleaned 300 bp dsDNA with the 4 base 5′ overhanging ends was created as above and treated with T4 ligase (New England Biolabs, cat. # M0202), either in the absence or presence of HMGB1 (Biolegend, cat. # 557804), using the manufacturer’s recommended protocol (24 h at 37 °C). Ligase reactions were stopped with Proteinase K treatment (1 h at 37 °C, New England Biolabs, cat. # P8111) and then cleaned using the QIAquick PCR clean-up kit (Qiagen, cat. # 28104). Linear species remaining in the sample were removed using either T5 or T7 exonuclease (New England Biolabs, cat. # M0363, M0263) using the manufacturer’s recommended protocol.

### Electrophoretic mobility shift assay

All recombinant proteins used for EMSA experiments were handled according to manufacturers’ recommendations. Human aSyn and pSyn proteins (Proteos, cat. # RP-003, RP-004) and human aSyn, bSyn, gSyn, A30P, E46K, A53T, deltaNAC aSyn (rPeptide, cat. # S-1001, S-1003, S-1007, S-1005, S-1008, S-1002, S-1015) were stored at −80 °C. Glutathione S-transferase (GenScript, cat. # Z02039) and DNA were stored at −20 °C. All DNA created using the 300 bp template DNA had 260/280 nm readings between 1.8 and 2.0, and 260/230 nm readings between 2.0 and 2.2. Protein–DNA incubation components were added in the following order: (1) buffer master mix, (2) recombinant protein, and (3) DNA. The final EMSA buffer solution contains 11 mM Tris, 1 mM EDTA, 50 mM NaCl, pH 8.0 to 8.5. Incubation occurred for 20 min on ice followed by 30 min at RT. For major and minor groove-binding dye experiments, incubations occurred for an additional 30 min at RT with the indicated DNA-binding dye (Hoechst 33258, Thermo Fisher, cat. # H1398; methyl green, Sigma-Aldrich, cat. # 198080). After the addition of 2 μl 10× Orange Loading Buffer (Li-Cor, cat. # 927-10100), samples were loaded into 6%, 10%, or 20% polyacrylamide Novex TBE gels (Invitrogen) and run in 1× TBE. All gels were poststained for 30 min at RT on a rotator with 1 to 10× Sybr Gold DNA Stain (Invitrogen, cat. # S11494) or Sybr Safe DNA Stain (Invitrogen, cat. # S33102) in 1× TBE. Images were acquired using a Fluorchem M (Protein Simple) imaging system and quantified using ImageJ (NIH) Gel Analyzing tool.

aSyn point mutation and deltaNAC EMSA protocol: rPeptide EMSA reactions contain 10 ng of a linear 300 bp fragment of DNA that was PCR amplified and cleaned up (Qiagen), 0.5% Ficoll 400 (Sigma cat. F4375), 4 μl of protein or buffer [20 mM Tris-HCl, 200 mM NaCl], and dH_2_O to a total volume of 10 μl. A master mix of 5% ficoll 400, DNA, and dH_2_O is added to a PCR reaction tube containing diluted protein and/or buffer. The reaction tube is gently flicked to mix. One microliter of LICOR 10× Orange loading dye (cat. 92710100) is added to the reaction and 8 μl loaded per lane of a Novex 10% TBE gel (cat. EC62755BOX). The gel is run in 0.5× TBE buffer (Corning, cat. 46-011-CM) at 100 V for 70 min, poststained in 1× SYBR Gold (Invitrogen cat. S11494) for 15 min, and imaged on a FluorChem ProteinSimple imager using the green 547 nm filter. The gel is then fixed for 30 min in 40% methanol, 10% acetic acid, followed by poststaining overnight with Biorad Bio-Safe Coomassie G-250 Stain (cat. 1610786). After washing in dH_2_O, the gel is imaged on a LICOR Odyssey in the 700 channel. Gels are analyzed using ImageJ (NIH) Gel Analyzing tool.

### Western blot and Coomassie stain

In total, 6% and 10% polyacrylamide TBE gels were transferred onto a Biodyne B Nylon Membrane (Thermo Fisher Scientific, cat. # 77016) at 30 to 35 V for 1 h and 15 min on ice in 0.5× TBE using the Novex X-Cell II Blotting System (Invitrogen). After transfer, membranes were washed briefly in H_2_O and fixed for 30 min at RT in Ca^2+^/Mg^2+^-free 1× PBS containing 4% paraformaldehyde and 0.1% glutaraldehyde. Postfixation, membranes were washed 3× (10 min each) in Ca^2+^/Mg^2+^-free PBS containing 0.1% Triton X-100. Membranes were blocked overnight in Odyssey PBS Blocking Buffer (Li-Cor, cat. # 927-40000) and stained for 1 h at RT with Syn1 primary antibody (1:1000; BD Biosciences, cat. # 610787) and overnight at 4 °C with IRDye 800CW Goat anti-Mouse IgG secondary antibody (1:10,000; Li-Cor, cat. # 926-32210). Images were acquired using Li-Cor Odyssey CLx Imaging System. After DNA imaging, some gels were washed 1× (5 min) in MilliQ H_2_O and stained in 15 to 20 ml of Coomassie G250 Safestain (Bio-Rad, cat. # 1610786) overnight at 4 °C on a rotator. Gels were washed 2× (1 h, then 2 h) in MilliQ H_2_O. Images were acquired using a Li-Cor Odyssey CLx Imaging System.

### Transmission electron microscopy

Five microliters of DNA suspensions was deposited onto glow discharged (120 s 15 mAmp, negative mode) carbon formvar 400 Mesh copper grids (Ted Pella 01822-F) for 3 min, rinsed 10 s in water, wicked on Whatman filter paper 1, stained for 3 min in freshly prepared 1% (w/v) uranyl acetate in water, wicked, and air-dried. Samples were imaged at 120 kV on a FEI Tecnai Spirit TEM system. Images were acquired using the AMT interface on an AMT 12 Megapixel NanoSprint12S-B cMOS camera system.

### Atomic force microscopy

Five nanograms of 300 bp linear DNA suspended in the same buffer used for the EMSA assay (see above), with the addition of 5 mM MgCl_2_, was deposited on a mica surface without the presence of aSyn. Similar experiments using 57 μM aSyn with 5 ng of 300 bp linear DNA (suspended in 3 mM HEPES at pH 8.0 with 10 mM NaCl, 2 mM MgCl_2_) were performed after deposition on silicon surfaces. AFM samples were prepared using 10 μl of the DNA solution. First, 5 μl of a 5 mM MgCl_2_ solution was deposited dropwise onto freshly cleaved mica or silicon and dried under a clean flux of N_2_ gas. This was followed by the deposition of 5 μl of the DNA-MgCl_2_ solution onto the surface, which was dried with N_2_ gas. The sample was then rinsed with NANOpure H_2_O to remove excess salt and loosely bound DNA and dried under N_2_ gas. The same procedure was used for samples containing alpha-synuclein and DNA that were allowed to incubate for 10 min before casting onto the surface. Imaging was performed using Digital Instruments Veeco AFM/LFM Instrument (Veeco Metrology group). Rotated monolithic, uncoated silicon AFM probes were used with 125 μm tip length, 300 kHz resonant frequency, and a 40 Newton/meter spring constant model Tap300-G (Ted Pella Inc). The instrument was operated in tapping mode while keeping drive amplitude to a minimum, collecting scans of 2 μm × 2 μm at a slow scan frequency of 1.5 to 2.5 Hz and 512 × 512 scans per line. AFM images were analyzed using Digital Instruments software.

### Experimental design and statistical analysis

All quantified values are reported as the mean ± SD. The relevant sample type, number (N), and statistical tests used to evaluate significance for each experiment are presented with each data set.

## Data availability

All data are presented in the article and the supplementary information.

## Supporting information

This article contains supporting information (40).

## Conflict of interest

The authors declare that they have no conflicts of interest with the contents of this article.
