# Supplementary figures and images for: Phosphorylation of the aggregate-forming protein alpha-synuclein on serine-129 inhibits its DNA-bending properties
Source: J Biol Chem. 2021 Dec 30;298(2):101552. doi: 10.1016/j.jbc.2021.101552 (PMC8800120; doi:10.1016/j.jbc.2021.101552)

A

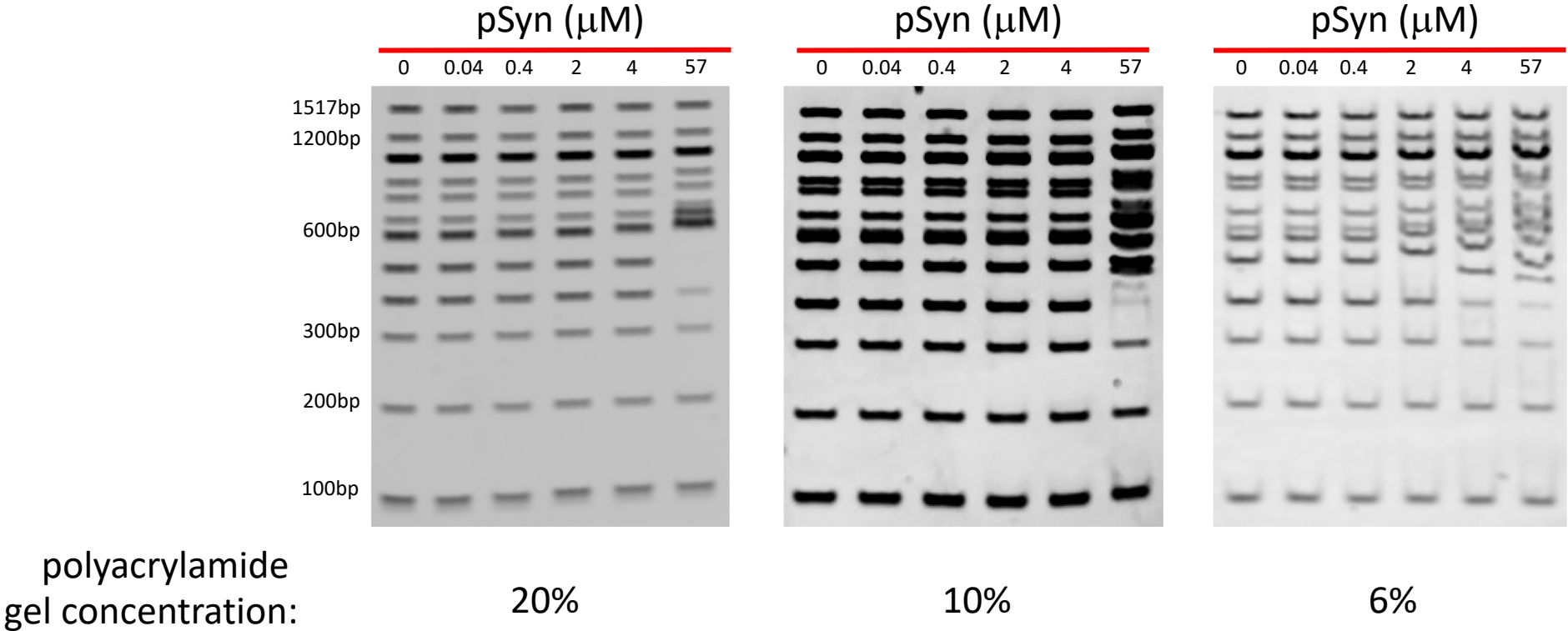

B

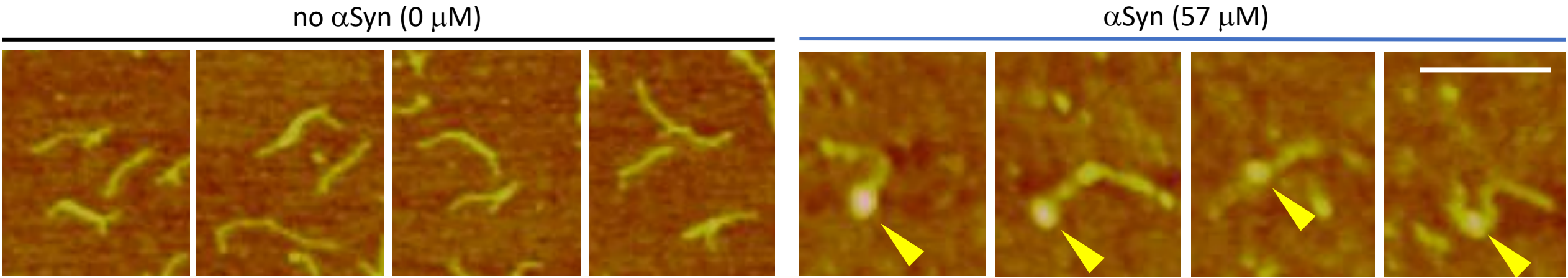

Supplement: Supplemental Figure S1 [file mmc1.pdf]

**A**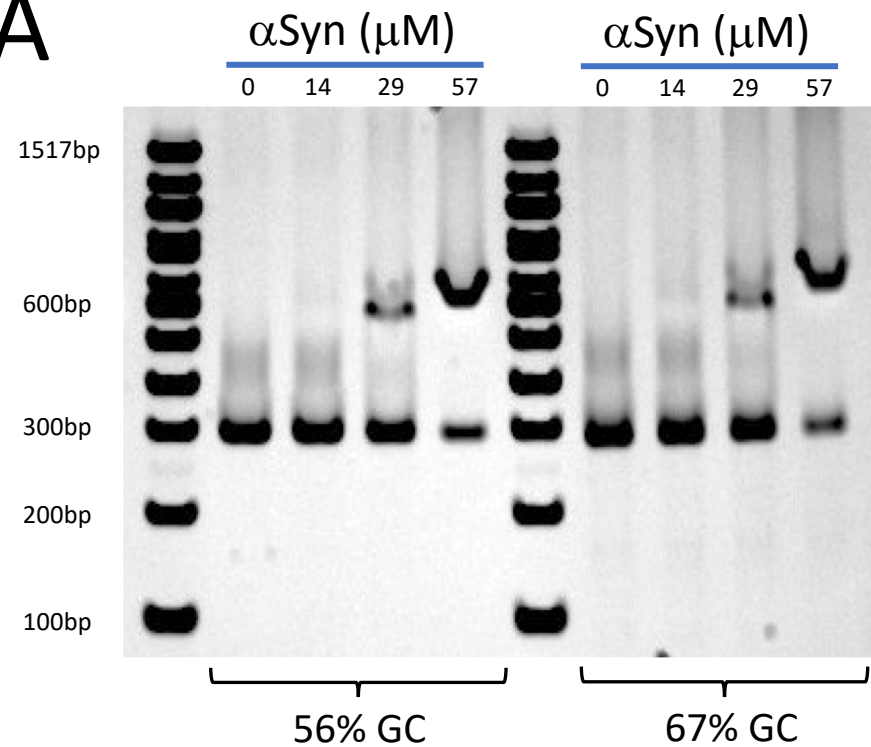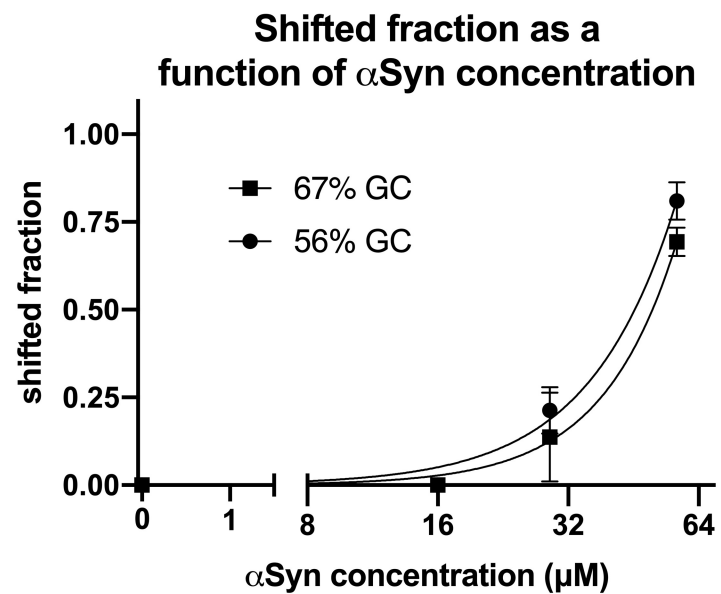**B**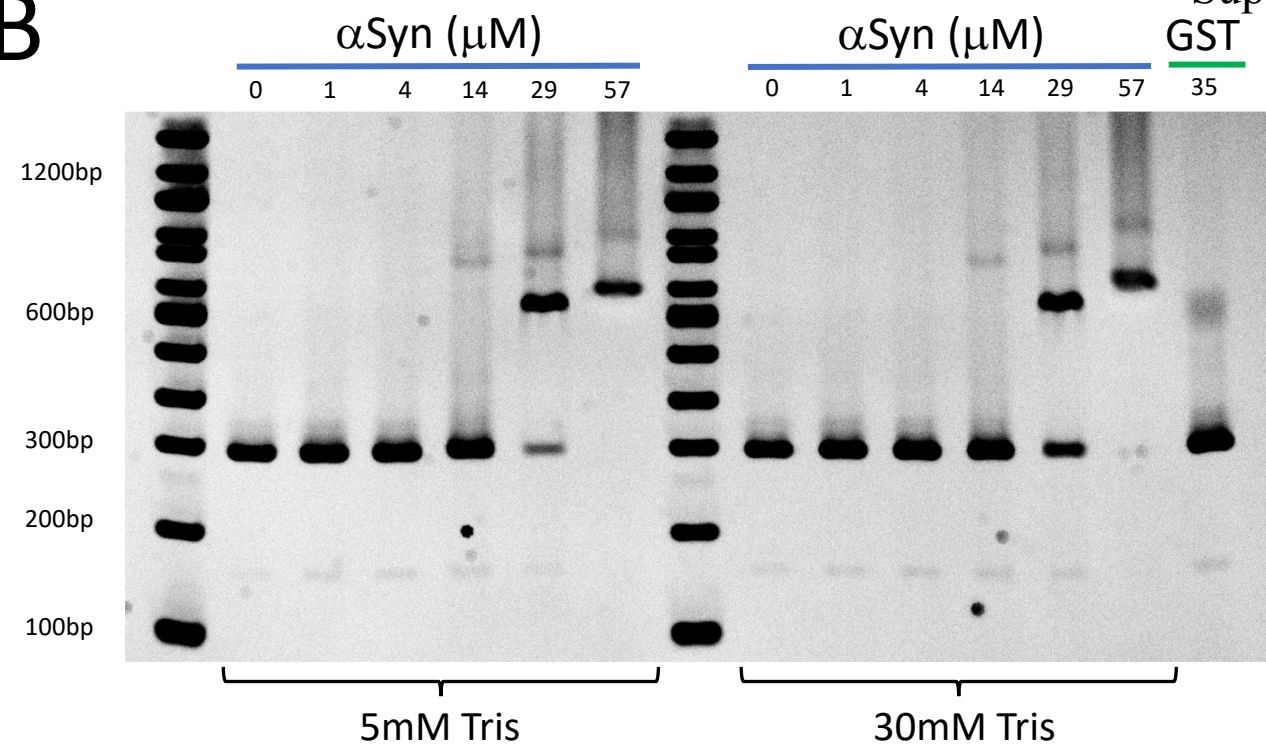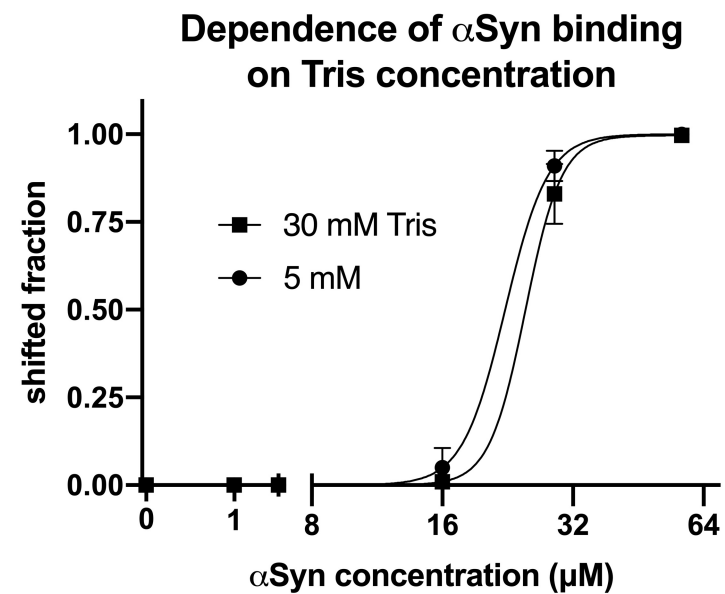

Supplement: Supplemental Figure S2 [file mmc2.pdf]

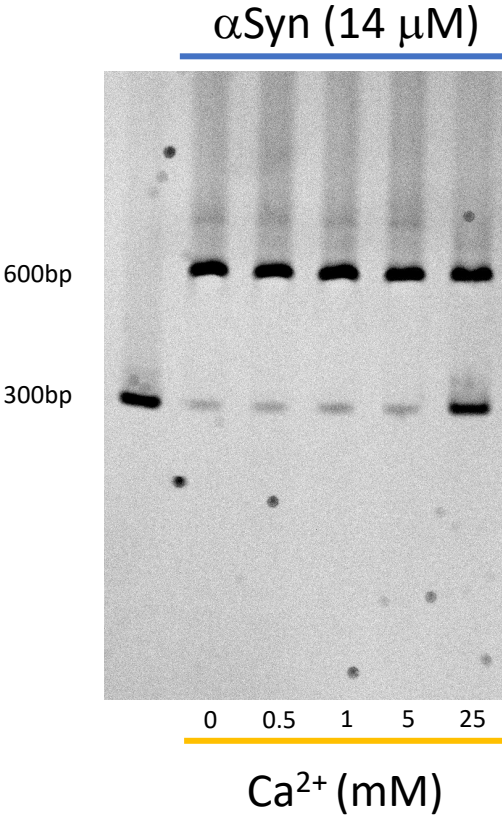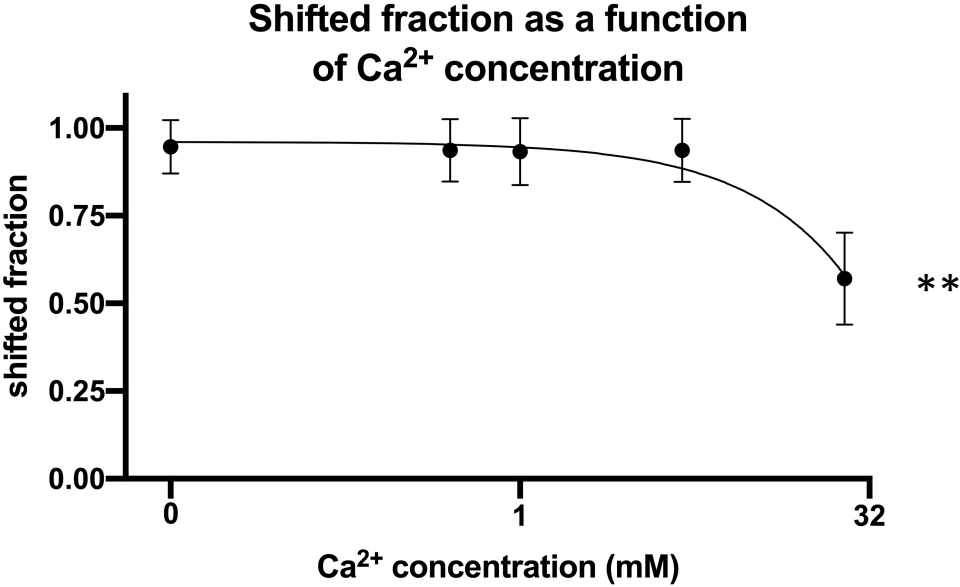

Supplement: Supplemental Figure S3 [file mmc3.pdf]

$\alpha$ Syn or pSyn  
(57  $\mu$ M):

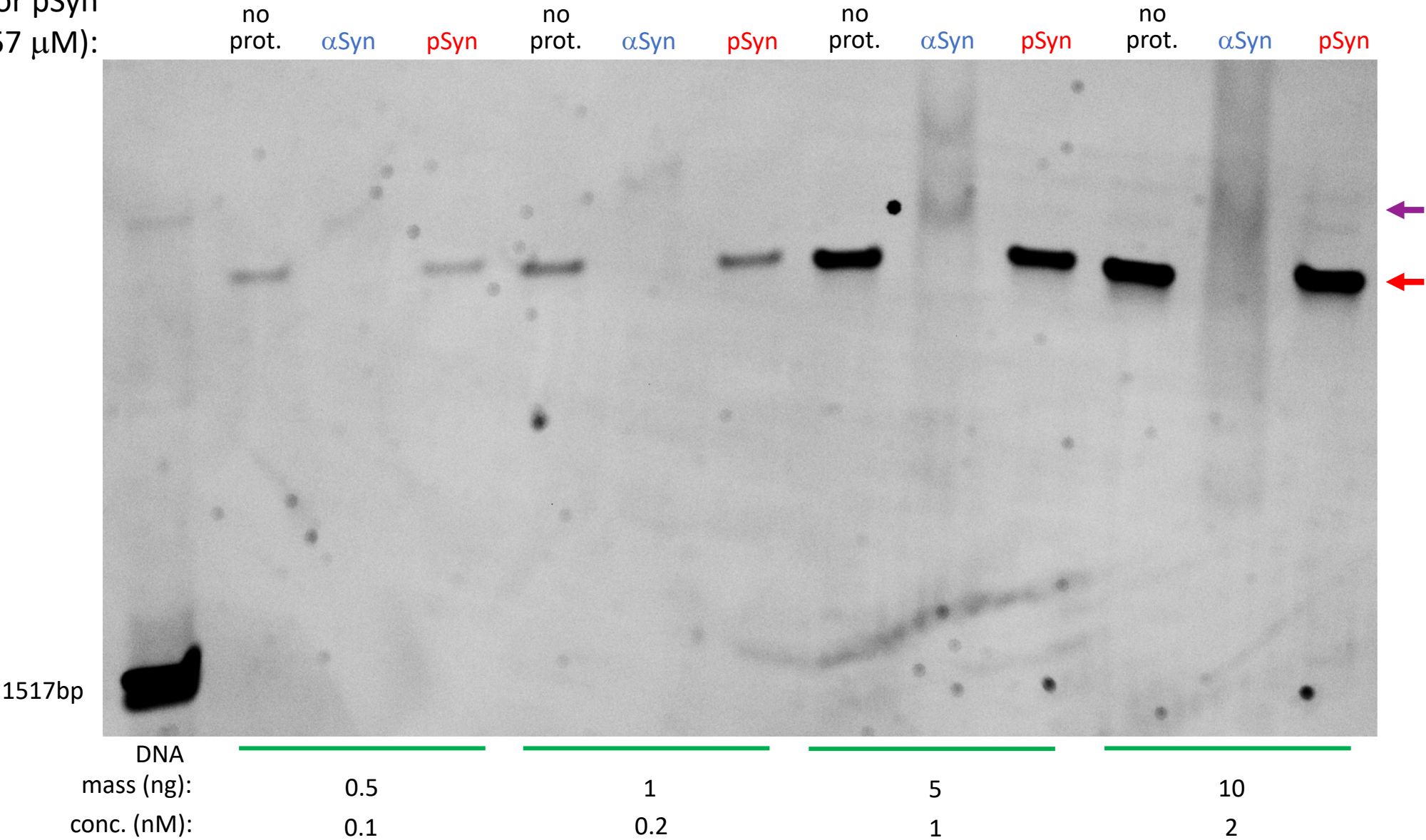

Supplement: Supplemental Figure S4 [file mmc4.pdf]

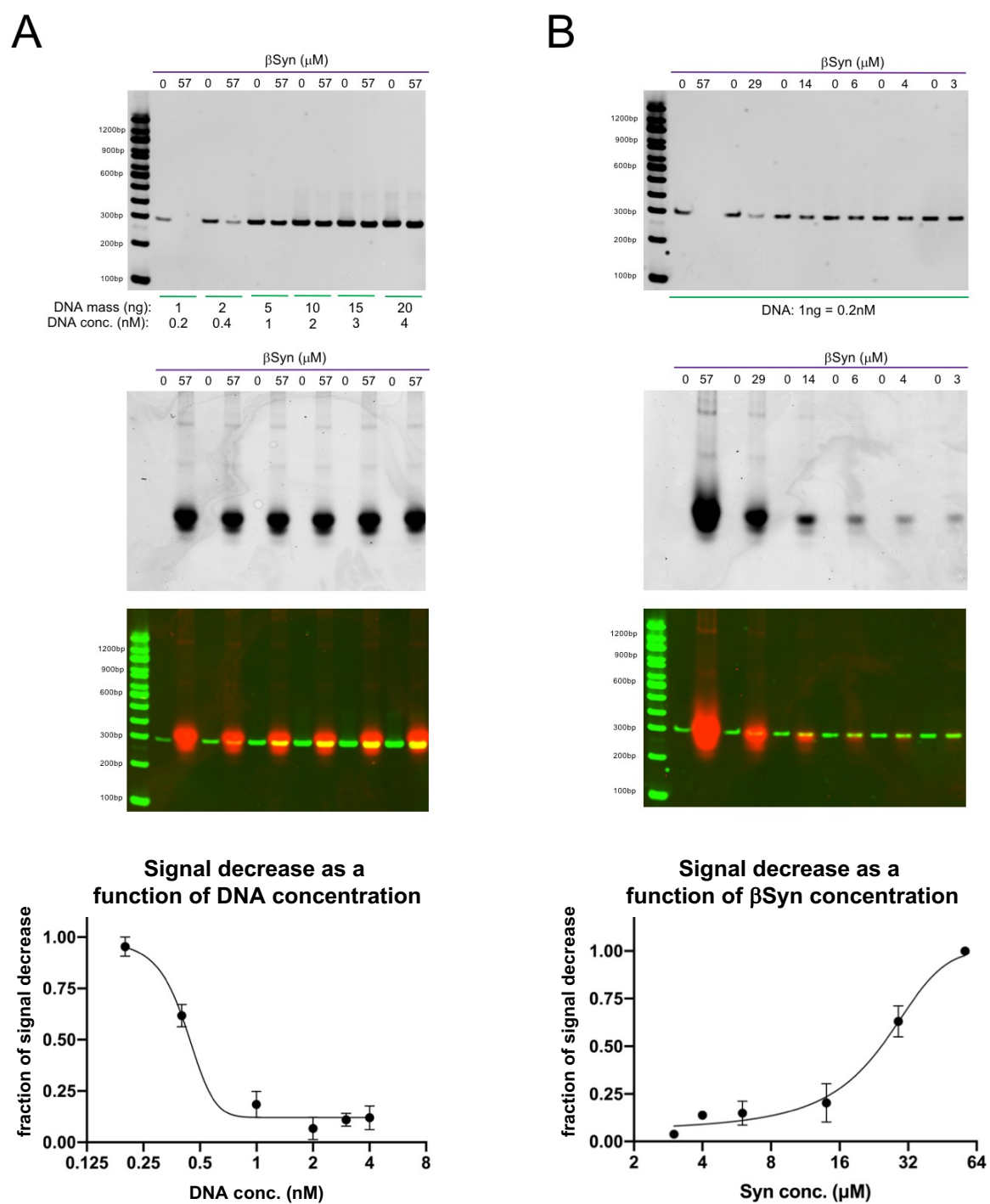

Supplement: Supplemental Figure S5 [file mmc5.pdf]

A

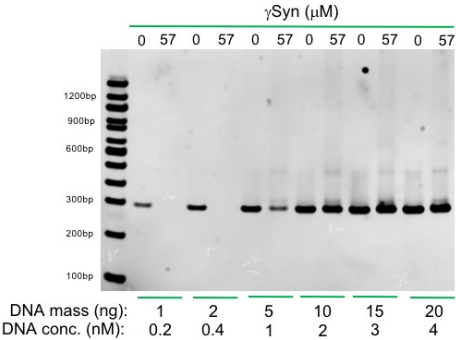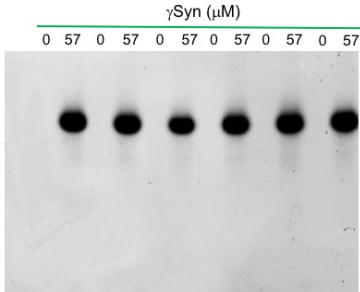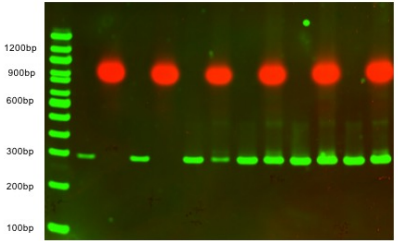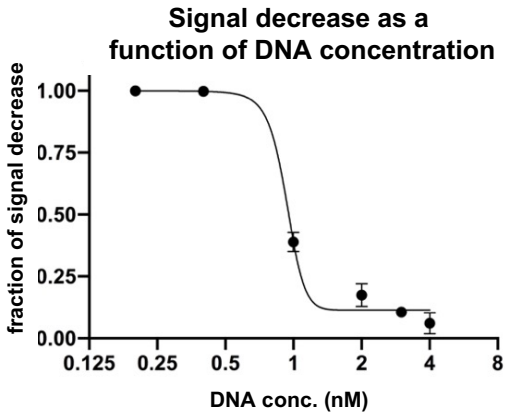

B

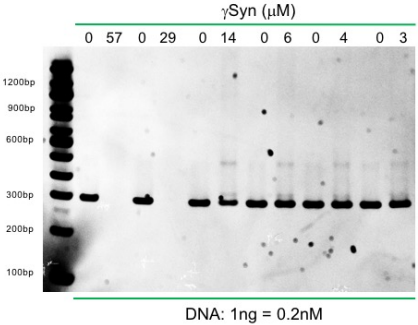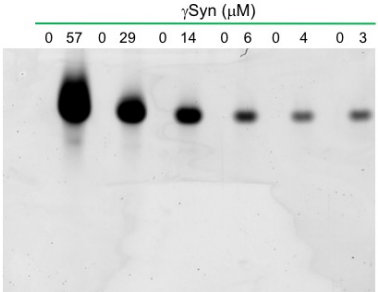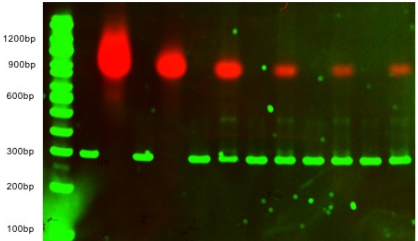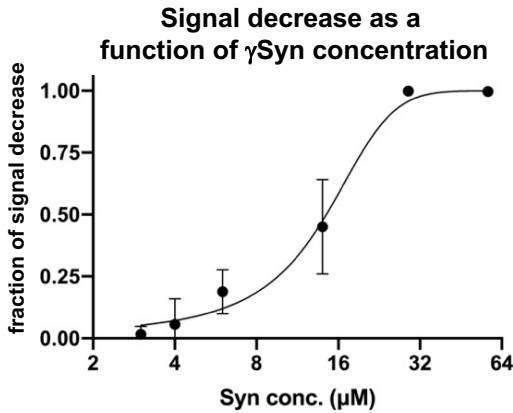

Supplement: Supplemental Figure S6 [file mmc6.pdf]
