# Supplementary material for: Phosphorylation of the aggregate-forming protein alpha-synuclein on serine-129 inhibits its DNA-bending properties
Source: J Biol Chem. 2021 Dec 30;298(2):101552. doi: 10.1016/j.jbc.2021.101552 (PMC8800120; doi:10.1016/j.jbc.2021.101552)
Supplement: Figure Legends [file mmc7.pdf]

SUPPLEMENTARY FIGURE LEGENDS

Supplementary Figure 1. The electrophoretic shift pattern of phospho-synuclein with DNA is similar under different gel conditions and atomic force microscopy shows evidence of alpha-synuclein binding to DNA. (**A**) Three different polyacrylamide gel concentration conditions show a shift of DNA of different lengths with increasing serine-129 phosphorylated alpha-synuclein (pSyn) concentration. (**B**) Atomic force microscopy (AFM) images of 300 bp linear DNA deposited on a surface without the presence of alpha-synuclein (aSyn, *left*), or in the presence of 57 μM aSyn (*right*). In the presence of aSyn, apparent DNA thickness is increased and a globular protein signal bound to DNA is often apparent (yellow arrowheads). Scale bar 200 μm.

Supplementary Figure 2. Alpha-synuclein-DNA binding is not strongly dependent on DNA sequence or Tris concentration. (**A**) Alpha-synuclein (aSyn) binds DNA of different sequences, containing either 56 or 67% GC content, similarly (shifted fraction 56% GC at 0, 14, 29 and 57 μM aSyn: 0.000±0.000, 0.000±0.000, 0.213±0.067, 0.810±0.053, Hill slope=2.2; shifted fraction 67% GC at 0, 14, 29 and 57 uM μSyn: 0.000±0.000, 0.000±0.000, 0.137±0.127, 0.693±0.040, Hill slope=2.5; comparison shifted fraction of 57 and 67% GC content DNA for each aSyn concentration by paired t-test shows no significant differences, p between 0.162-0.373; variable slope dose-normalized response curve; N=3 gels, x-axis aSyn concentration on log scale). (**B**) aSyn binds DNA at different Tris concentrations similarly (shifted fraction 5 mM Tris at 0, 1, 4, 14, 29 and 57 μM aSyn: 0.000±0.000, 0.000±0.000, 0.000±0.000, 0.050±0.056, 0.910±0.044, 1.000±0.000; shifted fraction 30 mM Tris at 0, 1, 4, 14, 29 and 57 μM aSyn: 0.000±0.000, 0.000±0.000, 0.000±0.000, 0.010±0.017, 0.830±0.085, 0.997±0.006; comparison shifted fraction of 5 and 30 mM Tris conditions for each aSyn concentration by paired t-test shows no significant differences, p between 0.150-0.423; 4-parameter dose-response curve; N=3 gels, x-axis aSyn concentration on log scale). Glutathione-S Transferase (GST) protein run as a negative control shows little shifted species.

Supplementary Figure 3. Alpha-synuclein-DNA binding is decreased by increasing calcium ion concentration. Increasing concentration of the divalent cation Ca^{2+} (0-25 mM) decreases alpha-synuclein (aSyn) binding to 300 bp DNA at the highest concentration (R^2=0.791, deviation from zero slope p=0.0024, 3-parameter dose-response curve; N=2 gels; x-axis Ca^{2+} concentration on log scale).

Supplementary Figure 4. Alpha-synuclein binds 304 bp circular DNA forms created without the presence of HMGB1 and phospho-synuclein does not. Polyacrylamide (6%) gel electrophoresis shows a single circular DNA form created when reacting linear DNA with T4 ligase and without the presence of the DNA-bending protein HMGB1 (red arrow). Only aSyn, and not pSyn, produces a shift to a higher apparent length of these DNA circles (purple arrow).

Supplementary Figure 5. Beta-synuclein binds DNA differently than alpha-synuclein. (**A**) *Top row:* Decreasing 300 bp DNA concentration from 4 to 0.2 nM (from right to left) with fixed beta-synuclein (bSyn) concentration (57 μM) produces a loss of DNA signal at high bSyn-to-DNA ratios. *Second row:* Coomassie stain showing bSyn protein loaded into each lane. *Third row:* DNA gel (green) and bSyn Commassie stain (red) localization from the same experiment. *Bottom row:* Group data showing loss of DNA signal as a function of DNA concentration (bSyn IC50=0.442 μM, R^2=0.973; absolute IC50 fit; N=3 gels, x-axis DNA concentration on a log scale). (**B**) *Top row:* Increasing bSyn protein concentration from 3 to 57 μM (increasing right to left) with 300 bp DNA concentration fixed at 0.2 nM also produces a loss of DNA signal at high bSyn-to-DNA ratios. *Second row:* Coomassie stain showing bSyn protein loaded into each lane. *Third row:* DNA gel (green) and bSyn Coomassie stain protein (red) localization from the same experiment. *Bottom row:* Group data showing loss of DNA signal as a function of bSyn concentration (bSyn EC50=24.38 μM, R^2=0.970, absolute EC50 fit; N=3 gels, x-axis bSyn concentration on log scale).

Supplementary Figure 6. Gamma-synuclein binds DNA similarly to beta-synuclein and differently than alpha-synuclein. (**A**) *Top row:* Decreasing 300 bp DNA concentration from 4 to 0.2 nM (from right to left) with fixed gamma-synuclein (gSyn) concentration (57 μM) produces a loss of DNA signal at high gSyn-to-DNA ratios. *Second row:* Coomassie stain showing gSyn protein loaded into each lane. *Third row:* DNA gel (green) and gSyn Coomassie stain (red) localization from the same experiment. *Bottom row:* Group data showing loss of DNA signal as a function of DNA concentration (gSyn IC50=0.952 μM, R^2=0.989; absolute IC50 fit; N=3 gels, x-axis DNA concentration on log scale). (**B**) *Top row:* Increasing gSyn protein concentration from 3 to 57 μM (increasing right to left) with 300 bp DNA concentration fixed at 0.2 nM also produces a loss of DNA signal at high gSyn-to-DNA ratios. *Second row:* Coomassie stain showing gSyn protein loaded into each lane. *Third row:* DNA gel (green) and gSyn Coomassie stain protein (red) localization from the same experiment. *Bottom row:* Group data showing loss of DNA signal as a function of gSyn concentration (gSyn EC50=14.46 μM, R^2=0.956, absolute EC50 fit; N=3 gels, x-axis gSyn concentration on log scale).

Supplemental Figure 7. Docking model of alpha-synuclein bound to double-stranded DNA. Full animation showing a model of aSyn bound to DNA. The aSyn coordinates come from previous work showing its structure bound to a phospholipid micelle (40). The two curved alpha-helices of the N-terminal domain of aSyn fit into consecutive major grooves of B form DNA. Lysine residues within the protein are labeled.

Supplemental Figure 8. Docking model of alpha-synuclein bound to double-stranded DNA. Full animation showing a model of aSyn bound to DNA. The aSyn coordinates come from previous work showing its structure bound to a phospholipid micelle (40). The first (most N-

terminal) 12 lysine residues are labeled. In this particular model, potential electrostatic interactions between lysine-21, -23, and -80 with the phosphate backbone of DNA are present.
